# Mammalian Epidermis: A Compendium of Lipid Functionality

**DOI:** 10.3389/fphys.2021.804824

**Published:** 2022-01-12

**Authors:** Matteo Vietri Rudan, Fiona M. Watt

**Affiliations:** Centre for Stem Cells and Regenerative Medicine, King’s College London, Guy’s Hospital, London, United Kingdom

**Keywords:** lipids, epidermis, keratinocytes, ceramides, signaling, fatty acids, lipidomics

## Abstract

Mammalian epidermis is a striking example of the role of lipids in tissue biology. In this stratified epithelium, highly specialized structures are formed that leverage the hydrophobic properties of lipids to form an impermeable barrier and protect the humid internal environment of the body from the dry outside. This is achieved through tightly regulated lipid synthesis that generates the molecular species unique to the tissue. Beyond their fundamental structural role, lipids are involved in the active protection of the body from external insults. Lipid species present on the surface of the body possess antimicrobial activity and directly contribute to shaping the commensal microbiota. Lipids belonging to a variety of classes are also involved in the signaling events that modulate the immune responses to environmental stress as well as differentiation of the epidermal keratinocytes themselves. Recently, high-resolution methods are beginning to provide evidence for the involvement of newly identified specific lipid molecules in the regulation of epidermal homeostasis. In this review we give an overview of the wide range of biological functions of mammalian epidermal lipids.

## Introduction

The evolution of an impermeable barrier that could preserve the internal aqueous environment of the body away from water in ancestral reptiles was one of the most important events that allowed the colonization of dry land by vertebrates. That barrier, the “cornified” or “horny” epidermis, leveraged the physical properties of hydrophobic lipid and protein molecules to achieve the separation between the “wet” inside and the “dry” outside. Since then, over the course of evolution, the epidermis has acquired numerous additional structures and adaptations. However, the core physical mechanisms through which it exerts its most fundamental functions as well as its basic architecture have remained similar in all terrestrial vertebrates (reptiles, birds and mammals).

**FIGURE 1:**
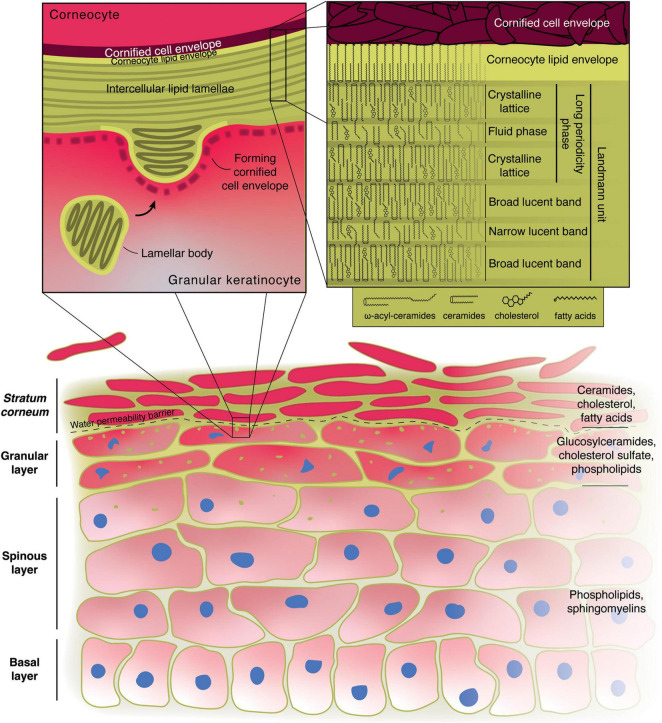
Role of lipids in the formation of the epidermal water permeability barrier. Illustration of the structure of the skin with the main lipid components of the various layers indicated on the right. The left inset shows the microstructure at the boundary between the granular layer and the *stratum corneum*. The right inset displays the possible molecular arrangement of the main *stratum corneum* lipids in the intercellular lamellae according to the “sandwich model.” Alternative models have also been proposed.

In mammals, the epidermis is the outermost part of the skin, overlying the connective tissue, the dermis. Mammalian epidermis is a stratified epithelium comprising a series of layers of progressively more differentiated cells, called keratinocytes. At the dermal interface, resting on a basement membrane, the basal keratinocyte layer contains cycling cells, responsible for the renewal of the tissue. Basal cells undergo a phase of commitment and then start the process of differentiation, exiting the cell cycle and beginning to migrate upward toward the body surface. As the cells progress through differentiation, they move through the spinous layer and the granular layer, all the while accumulating specific lipids and proteins and building functional ultrastructures. The keratinocytes ultimately lose their nucleus and become flattened “bricks” of insoluble protein called corneocytes, surrounded by lipid “mortar” to hold them together in the outermost impermeable cornified layer or *stratum corneum* (Nemes and Steinert, 1999; Watt, 2014; Figure 1).

Lipids are essential for the fundamental function of the epidermis since they are a key element in the water-insulating properties of the cornified layer. The relative abundance of different lipid species changes across the various layers of the epidermis, underscoring how the correct establishment of a functional barrier requires tightly controlled regulation of lipid production (Lampe et al., 1983). Indeed, dysregulation of the lipid balance can cause a number of pathological conditions, notably ichthyoses (Akiyama, 2017; Vahlquist and Törmä, 2020).

Beyond their structural role in the formation of the physical barrier of the epidermis, several studies have unraveled the participation of lipid molecules in the active protection of the body from external harmful agents, such as pathogenic microbes and damaging UV-radiation. Moreover, several studies have shown how specific lipids may be directly involved in the regulation of keratinocyte differentiation itself, and new techniques are now allowing the exploration of this field of research.

This review covers the multi-faceted roles, from structural to regulatory, that lipids play in epidermal homeostasis and protection from the external environment. The epidermis is a model of the versatility of this class of molecules, highlighting their important, often understated, and still potentially uncharacterized activities. We first describe the central role of lipids in the formation of the epidermal water permeability barrier. Then we detail the antimicrobial and immunomodulatory activity of certain epidermal lipids. Finally, we analyze how epidermal lipids of many different classes can influence epidermal cell signaling. This review will not, however, cover lipid vitamins. For details of the extremely important part vitamin A, vitamin D and their derivatives play in the biology of the epidermis, the reader is referred to recent reviews (Piotrowska et al., 2016; Szymański et al., 2020).

## The Body’s Outer Wall – the Fundamental Structural Role of Epidermal Lipids

The lipid composition varies dramatically along the thickness of mammalian epidermis (Figure 1). Early dissections of the lipid makeup of the epidermis of different mammals revealed striking differences between the basal and spinous layers, the granular layer and the cornified layer (*stratum corneum*) (Kooyman, 1932; Long, 1970).

### Basal and Spinous Layers

In the basal and lower spinous layers, phospholipids are most common, with high levels of phosphatidylcholines, phosphatidylethanolamines, phosphatidylserines and sphingomyelins (Gray et al., 1980). This phospholipid predominance likely underlies the main roles that lipids have in these cells: membrane maintenance, energy production and signaling.

### Granular Layer

As keratinocytes progress through differentiation and become more specialized, their lipid and protein makeup change. Starting from the upper spinous layer and in the granular layer the keratinocytes begin forming cross-linked bundles of keratin fibers and filaggrin in their cytoplasm. Their organelles begin degenerating and they assemble lamellar bodies (LB), also known as membrane-coating granules, lamellar granules, or Odland bodies (Selby, 1957; Odland, 1960; Proksch et al., 2008; Wertz, 2018). LB have been traditionally described as membrane-bound organelles of ∼200 nm in diameter containing a series of bilayer membranes 6–7 nm thick that are closely stacked together (Matoltsy and Parakkal, 1965; Figure 1), although more recent evidence suggests that they form a tubuloreticular network derived from the trans-Golgi apparatus (Matoltsy and Parakkal, 1965; Elias et al., 1998; Norlén et al., 2003).

Early characterization of the contents of LBs revealed that they are mostly made up of lipids and are specifically rich in glycolipids (mostly glucosylceramides), phospholipids and cholesterol as well as smaller amounts of sterol esters, ceramides, and fatty acids (Wertz et al., 1984; Grayson et al., 1985). An especially remarkable lipid species found uniquely in LB is an acylglucosylceramide comprising a linoleic acid (C18:2ω6) molecule esterified to the ω-hydroxy group of a very long chained (30–34 carbons) fatty acid moiety of the glucosylceramide (Wertz et al., 1984; Bowser et al., 1985). A specific role has been proposed for this particular lipid in the correct structural assembly of LBs, whereby its very long ω-hydroxyacid chain is able to span an entire lipid bilayer while the linoleate tail can insert itself in a neighboring bilayer, thus acting as a “molecular rivet” holding together the tightly packed series of membranes found in LBs (Wertz and Downing, 1982; Wertz, 2018). Importantly, besides its potential role in structuring the LB’s contents, the linoleic acid-containing acylglucosylceramide is an essential component of the LB’s bounding membrane, where the majority of it is found (Wertz, 2000, 2018).

Besides lipid molecules, LBs contain several hydrolytic enzymes such as carboxypepdidase, cathepsin B, acid hydrolase, as well as acid lipase, β-glucosidases, phospholipase A, sphingomyelinase, ceramidases, and steroid sulfatase that play an important role during the subsequent differentiation steps (Freinkel and Traczyk, 1985; Grayson et al., 1985).

### Cornified Layer (*Stratum corneum*)

The boundary between the granular and cornified layers represents the water-permeability barrier of the body, as demonstrated by dermal injection of water-soluble tracers (Elias and Friend, 1975). As the cells move toward and through this boundary, they become corneocytes, the “bricks” of the *stratum corneum*: they form an extremely resistant protein shell, the cornified cell envelope, just beneath their plasma membrane, made of crosslinked involucrin, loricrin, small proline-rich proteins and other proteins. The nucleus starts to degenerate and ultimately disappears, as the cytoplasm becomes filled with keratin bundles and the cell becomes flattened. The LBs approach the apical plasma membrane of the cell, their bounding membrane finally fusing with it, releasing the LB contents in the extracellular space (Wertz, 1996; Proksch et al., 2008; Figure 1).

The linoleate-containing acylglucosylceramide in the bounding membrane is processed to become ω-hydroxyceramide, which is covalently bound to the cornified cell envelope’s proteins, notably involucrin, forming an additional layer around the cell, known as the corneocyte lipid envelope (Swartzendruber et al., 1987; Wertz and Downing, 1987; Nemes et al., 1999; Grond et al., 2017). Together, the cornified cell envelope and the lipid envelope replace the plasma membrane of the corneocyte.

**FIGURE 2:**
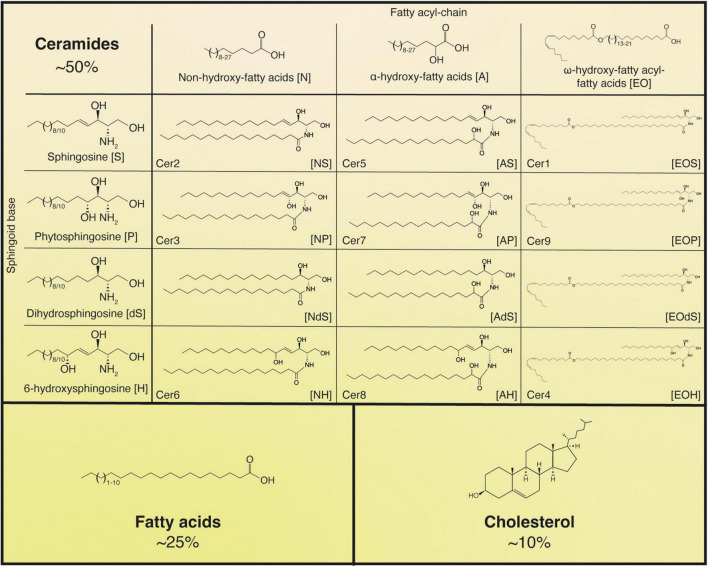
Main lipids of the *stratum corneum*. Ceramide classes are labeled both according to the classical nomenclature (Cer1-9) when applicable and by the letter code indicating the sphingoid base-fatty acyl moiety combination. In the example structures of the ceramides, all sphingoid bases and fatty acid moieties are shown as C18, but can be substituted by other chain lengths according to what is indicated at the top of the figure or described in the main text. Cer[EO] structures are presented as C26 fatty acids esterified to linoleic acid (as that is what is most commonly found in the epidermis). Both ceramide fatty acyl groups and *stratum corneum* free fatty acids are generally saturated or monounsaturated.

Upon extrusion, the lipids contained in the LBs undergo dramatic changes, likely becoming substrates for the lipid hydrolases that convert them into ceramides (∼50%), cholesterol (∼25%), and fatty acids (∼10%), the main constituents of the intercellular lipid “mortar” of the cornified layer (Figure 2), which also comprises smaller amounts of cholesterol sulfate and cholesterol esters (Nicolaides, 1974; Wertz and van den Bergh, 1998). Notably, the release of fatty acids from phospholipids by phospholipase A_2_ contributes to the creation of an acidic environment in the *stratum corneum*, which in turn regulates the activity of other enzymes, such as β-glucocerebrosidase, acid sphingomyelinase as well as serine proteases. This is necessary to ensure the correct formation of the epidermal barrier and its homeostatic regulation (Hachem et al., 2003).

The chemical changes in lipids are accompanied by morphological ones, as the disk-like bilayers seen in the LBs fuse to become a series of broad lamellar sheets (Elias et al., 1977; Landmann, 1986; Madison et al., 1987; Figure 1). The corneocyte lipid envelope plays a fundamental role in the assembly of these lamellae, acting as a template for their orientation with respect to the corneocytes (Behne et al., 2000). More specifically, lipids arrange themselves into repeating units that appear as a series of broad-narrow-broad electron-lucent bands in electron micrographs. Each of these units (sometimes referred to as Landmann units) are thought to be juxtaposed lipid bilayers with an intervening monolayer formed by the long chains of the acylceramides acting as a zipper (Swartzendruber et al., 1989). X-ray diffraction studies of the human cornified layer’s intercellular lipid matrix instead describe two lamellar phases, referred to as the long-periodicity phase (LPP, ∼13 nm) and the short-periodicity phase (SPP, ∼5 nm), with the former likely to be relevant for the barrier function, having been identified in multiple animal species (Bouwstra et al., 1991, 1992). The LPP has in fact been equated to one Landmann unit, in which the ceramides, cholesterol and fatty acids in the outer bilayers arrange themselves as a dense crystalline orthorhombic lattice, responsible for hindering the movement of compounds through the outer epidermis, and form the impermeable barrier that lines the body, while the intervening monolayer made up of the ω-esterified unsaturated fatty acid (mostly linoleate) and cholesterol forms a liquid phase that could provide flexibility and resistance to shear stress. This is referred to as the “sandwich model” (Bouwstra et al., 2000, 2002; Figure 1).

Once the corneocytes reach the surface of the body, they are detached from the epidermis by desquamation. This process is mediated by the action of certain serine proteases that attack the desmosomes that connect corneocytes together (Suzuki et al., 1993). Cholesterol sulfate present in the *stratum corneum* acts as an inhibitor of these proteases. The concentration of cholesterol sulfate is highest in the granular layer, and from there it gradually decreases along the thickness of the cornified layer due to the action of steroid sulfatase. The lower concentration of cholesterol sulfate near the surface thus allows the proteases to become active and enables the eventual desquamation of the corneocytes (Elias et al., 1984; Sato et al., 1998).

### Heterogeneity of *Stratum corneum* Lipids

While the acylceramides, uniquely found in the epidermis, are crucial for the establishment of the water permeability barrier, there is considerable heterogeneity in the pool of *stratum corneum* ceramides and fatty acids, which is likely to contribute to the proper architecture of the lipid matrix (Figure 2).

Ceramides show a remarkably wide range of diversity due to heterogeneity in each of their sub-components. One of four different sphingoid bases – sphingosine, dihydrosphingosine, phytosphingosine, 6-hydroxysphingosine, usually C18 or C20 in length – can be paired with fatty acids that vary in carbon chain size (C15 to C34, though most are between C20 to C30), hydroxylation profile (nonhydroxy, α-hydroxy- or ω-hydroxy-), and presence of an ω-esterified fatty acid moiety (almost always linoleic acid) (Lampe et al., 1983; Masukawa et al., 2009; van Smeden et al., 2011). Based on the different combinations of these components, epidermal ceramides have been categorized into several classes (Figure 2). Free fatty acids of the cornified layer are nearly all saturated but their carbon chain length can vary between C18 and C28, with the most abundant species being C22 and C24 (Nicolaides, 1974; Wertz et al., 1987; Wertz and van den Bergh, 1998).

The functional importance of such lipid complexity is underscored by several studies that attempt to replicate the ultrastructure of cornified layer lipid lamellae in chemically defined model membranes (Bouwstra et al., 2002; de Jager et al., 2003; Opálka et al., 2016; Školová et al., 2017; Schmitt et al., 2019; Beddoes et al., 2021). It is further revealed by a variety of ichthyotic skin conditions in which different lipid biosynthetic pathways are perturbed (Akiyama, 2017; Vahlquist and Törmä, 2020).

## Beyond the Mortar – Lipids as Active Protectors of Epidermal Integrity

Besides its role as an impermeable physical barrier between the internal environment of the body and the outside world, the epidermis also actively participates in the protection of the organism against potential threats from opportunistic pathogenic micro-organisms (Natsuga et al., 2016; Fischer, 2020). Given its position as the outer boundary of the organism, the epidermal surface is host to a diverse set of commensal microbes, whose composition varies at different body sites, depending on certain physiological characteristics such as degree of moisture or abundance of sebaceous secretions (Table 1; Grice et al., 2009; Grice and Segre, 2011). Lipids produced in the epidermis can directly target surface-dwelling microbes to shape the commensal microbiota and quell the growth of potentially pathogenic species as well as participate in regulation of the innate and adaptive immune responses that follow after invasion of foreign pathogens.

**TABLE 1:** Main members of epidermal surface bacterial communities at different body sites grouped based on their microenvironmental characteristics.

Moist (e.g., armpit)	Sebaceous (e.g., glabella)	Dry (e.g., forearm)
*Corynebacterium* spp. (28%)	C*utibacterium* spp. (46%)	Betaproteobacteria (32%)
Betaproteobacteria (22%)	*Staphylococcus* spp. (16%)	*Corynebacterium* spp. (15%)
*Staphylococcus* spp. (21%)	*Corynebacterium* spp. (10%)	Flavobacteriales (14%)
Flavobacteriales (9%)	Betaproteobacteria (9%)	*Cutibacterium* spp. (13%)
*Cutibacterium* spp. (7%)	Flavobacteriales (3%)	Gammaproteobacteria (7%)
Gammaproteobacteria	Lactobacillales (3%)	*Staphylococcus* spp.
Lactobacillales	Clostridiales	Lactobacillales

*Bacteria are in order of abundance, with approximate average percentages in each microenvironment indicated. Data adapted from Grice et al. (2009).*

### Antimicrobial Lipids

The surface of the epidermis is a generally inhospitable environment for microbes owing to its mild acidity (pH 4-5.5), scarcity of water and poor availability of essential metabolites like phosphate (Aly et al., 1972, 1975; Forslind et al., 1995). Moreover, during differentiation, keratinocytes produce and secrete peptides and proteins that possess antimicrobial activity, such as defensins, cathelicidins (LL-37 in humans) and RNAse 7. Production of these peptides is stimulated upon damage to the epidermis (Fulton et al., 1997; Dorschner et al., 2001; Falconer et al., 2001).

**FIGURE 3:**
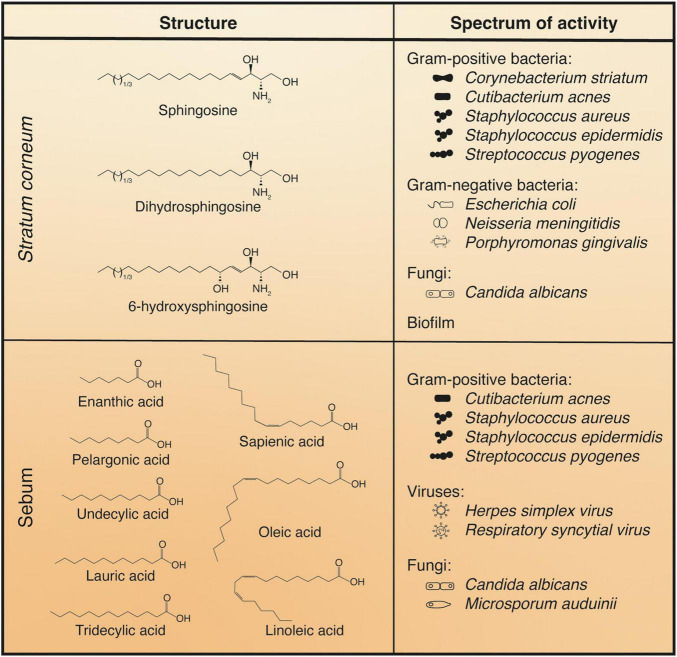
Main antimicrobial lipids of the epidermis. Rough spectra of activity are indicated on the right with a few examples. The lists of target micro-organisms are not exhaustive and efficacy can vary among the different *stratum corneum* sphingoid bases or the various sebaceous fatty acids.

In addition to peptides, one of the main antimicrobial components of the epidermis resides in its lipid fraction (Burtenshaw, 1942). Indeed, two major components of lipid-mediated immunity have been identified: free sphingoid bases in the *stratum corneum* and free fatty acids in the sebum (Figure 3).

#### Free Sphingoid Bases

One source of antimicrobial lipids comes from the *stratum corneum* lipids themselves. Among the enzymes contained in LBs are ceramidases, which cleave *stratum corneum* ceramides into a sphingosine base and a fatty acid. The fatty acids generated from these ceramides and more generally all *stratum corneum* fatty acids synthesized by keratinocytes have very long, mostly saturated carbon chains and do not possess any antiseptic activity (Rothman et al., 1946; Kabara et al., 1972, 1977; Zheng et al., 2005; Brogden et al., 2011). However, multiple studies have demonstrated the antimicrobial potency of sphingosine, dihydrosphingosine, and 6-hydroxysphingosine, which are found in the outer epidermis (Figure 3; Bibel et al., 1992). Phytospingosine also displays similar properties (Pavicic et al., 2007), but it is not found in free form in human *stratum corneum* (Wertz and Downing, 1990). The spectrum of antimicrobial activity of sphingosines is quite broad, including activity against numerous Gram-positive bacteria such as *Cutibacterium acnes*, *Staphylococcus aureus*, and *Streptococcus pyogenes*; some Gram-negative bacteria are also affected, such as *Escherichia coli* and *Porphyromonas gingivalis;* finally, fungi such as *Candida albicans* can also be targeted by these compounds (Figure 3; Bibel et al., 1992, 1993; Fischer et al., 2012b). The varying degrees of efficacy exhibited by sphingoid bases against different micro-organisms point to a certain level of specificity in their mode of action. In addition to their direct effect on bacterial growth and survival, sphingosines have proven very effective at interfering with bacterial biofilm formation (Seitz et al., 2019; Beck et al., 2020).

The mechanisms through which sphingoid bases enact their antimicrobial activity have not been fully elucidated. Treatment of *S. aureus* and *E. Coli* with sphingosine or dihydrosphingosine causes shrinking and distortion of the cells with alterations of cell membranes and emergence of inclusion bodies. In the Gram-positive *S. aureus*, the cell wall is lost and L-form strains, in which the cell wall is not synthesized, are more resistant to sphingoid base treatment. Interestingly, the cell wall of the Gram-negative *E. Coli* is not affected, implying that the effect on cell wall biosynthesis is likely a secondary consequence of the treatment. Given that the sphingoid bases become incorporated into the bacteria, one possibility is that they may insert themselves into the bacterial envelope/plasma membranes and render these structures non-functional. Alternatively, they may enter the cytoplasm and accumulate intracellularly where they might interfere with cellular metabolism (Bibel et al., 1993; Fischer et al., 2012b). This latter possibility is supported by the fact that sphingosines can participate in cellular signaling by inhibiting protein kinase C (PKC) in mammalian cells (Hannun et al., 1986). PKC is an important hub of cellular signaling; the different PKC isoforms vary in their regulation and can play numerous roles in influencing cellular behavior. Some PKC isoforms can be directly regulated by lipids such as diacylglycerol, as described below.

#### Sebaceous Free Fatty Acids

Another important source of antimicrobial lipids is sebum, a liquid mixture of neutral lipids assembled and secreted by sebaceous glands that coats and lubricates the outer epidermis. The function of sebum is to a degree still debated, but it has been implicated in helping to maintain the integrity of the epidermal barrier, in thermoregulation, photoprotection, and helping to deliver vitamin E to the skin (Zouboulis, 2004). Alterations in sebaceous secretions can lead to imbalances in the skin microbiome and are linked to pathological conditions. More specifically, the mutual influence between sebum and the commensal microbiota is exemplified by the fact that sebum-rich areas of the body host a specific subset of microbial species (Table 1), by the direct influence of bacterial lipases on sebum composition, and by the changes in sebum composition observed in acne patients (Scheimann et al., 1960; Lovászi et al., 2017; Dréno et al., 2020; Oulès et al., 2020).

Sebum composition is species-specific. The main components in humans are triglycerides (∼45%), wax esters (∼25%), squalene (∼12%), and fatty acids (∼10%). In contrast, mouse sebum is mostly made up of wax esters (∼70%), with a much smaller fraction of triglycerides (∼6%) and no fatty acids or squalene (Wilkinson and Karasek, 1966; Nicolaides et al., 1968; Nikkari, 1974; Pappas, 2009). Among human sebum components, fatty acids are the ones responsible for the antiseptic action of sebum. As sebum flows through the pilosebaceous duct, fatty acids are released from sebaceous triglycerides by the action of bacterial and possibly host lipases (Nicolaides and Wells, 1957; Scheimann et al., 1960; Marples et al., 1971; Götz et al., 1998; Zouboulis et al., 1999; Drake et al., 2008). When compared to those found in the *stratum corneum*, sebum fatty acids have shorter, in some cases odd-numbered, carbon chains, a higher degree of unsaturation, and display potent antimicrobial activity (Weitkamp et al., 1947). Among these, enanthic (C7:0), pelargonic (C9:0), undecylic acid (C11:0), and tridecylic acids (C13:0) extracted from human hair fat have antifungal activity that is implicated in protection against ringworm of the scalp (Rothman et al., 1946). Undecylenic acid (C11:1) is found in sweat and has widespread use as an antifungal treatment (Landau, 1983). Oleic (C18:1) and linoleic (C18:2) acid are abundant in sebum and have efficacy as antibacterials (Weitkamp et al., 1947; Kabara et al., 1972). Some of the most potent sebaceous fatty acids include lauric acid (C12:0), present in relatively minor amounts, and sapienic acid (C16:1ω10), thus named because it is the most abundant fatty acid found in *Homo sapiens* sebum (Figure 3; Weitkamp et al., 1947; Downing and Strauss, 1974; Stewart and Downing, 1991).

Sebum fatty acids tend to be more active toward Gram-positive bacteria (e.g., *C. acnes*, *S. aureus*) and lack efficacy against Gram-negative bacteria (e.g., *E. coli*, *P. aeruginosa*). This has been associated with the fact that the lipopolysaccharide-coated outer membrane of Gram-negative bacteria represents an effective barrier against penetration of hydrophobic compounds (Nikaido, 1976; Greenway and Dyke, 1979). Sebaceous fatty acid species can also be effective against certain viruses (Figure 3). Interestingly, the antimicrobial spectra of lauric and sapienic acids do not completely overlap (Kabara et al., 1972, 1977; Bergsson et al., 2001; Wille and Kydonieus, 2003; Thormar and Hilmarsson, 2007; Nakatsuji et al., 2009; Fischer et al., 2012a; Huang et al., 2014). As is the case with sphingosines, the variation in effectiveness of different compound-bacterium pairings may indicate some specificity in their action.

The molecular basis of the antimicrobial activity of sebaceous fatty acids is not understood completely (Desbois and Smith, 2010). The amphipathic nature of fatty acids is necessary for their antibacterial action, as replacing the -OH group at one end of the molecule with a methyl group abolishes any effect (Kodicek and Worden, 1945; Kabara et al., 1972; Zheng et al., 2005). The shape of the fatty acid molecule appears to affect its activity, with key determinants being carbon chain length as well as presence, number, position, and orientation of unsaturations. More specifically, for saturated species there is a tendency for antimicrobial potency to be highest at C10–12 and decrease with either longer or shorter carbon chains (Kabara et al., 1972; Bergsson et al., 2001; Wille and Kydonieus, 2003). The presence and number of unsaturations tend to increase efficacy at a given carbon length, with the *cis-* orientation (which is most often found in endogenous compounds) most effective in boosting activity (Kabara et al., 1972; Galbraith and Miller, 1973; Saito et al., 1984; Knapp and Melly, 1986). For example, despite its relatively long carbon chain, the double bond in sapienic acid is an unusual *cis* C-6 unsaturation that allows the molecule to adopt a conformation resembling a shorter-chained fatty acid (Fischer, 2020).

The principal target of sebum fatty acids seems to be the bacterial cytoplasmic membrane, where they are thought to interfere with oxidative phosphorylation-mediated energy production by way of disruption of the electron transport chain or dissipation of the membrane potential necessary for ATP synthesis (Sheu and Freese, 1972; Galbraith and Miller, 1973; Greenway and Dyke, 1979). One hypothesis is that the shorter-chained or *cis-* unsaturated fatty acids cause membrane fluidification and destabilization of electron transport proteins (Greenway and Dyke, 1979; Chamberlain et al., 1991). Other proposals are that they increase membrane permeability to protons (Borst et al., 1962; Greenway and Dyke, 1979; Gutknecht, 1988), or inhibit components of the ATP synthase machinery (Wojtczak and Załuska, 1967). Besides their action at the cell membrane, unsaturated fatty acids can inhibit cellular enzymes such as those responsible for nutrient uptake (Galbraith and Miller, 1973), and can inhibit fatty acid biosynthesis (Zheng et al., 2005; Sado-Kamdem et al., 2009). They can increase oxidative stress after undergoing peroxidation (Knapp and Melly, 1986) or undergo auto-oxidation yielding other antibacterial compounds (Gutteridge et al., 1974). Finally, fatty acids can cause leakage of intracellular components and cell lysis (Galbraith and Miller, 1973; Greenway and Dyke, 1979; Carson and Daneo-Moore, 1980). Understanding which of these mechanisms is responsible for the antimicrobial action of fatty acids is challenging, because some are connected (e.g., the decrease in energy production and the suppression of nutrient uptake, or the effect on membrane fluidity and the inhibition of fatty acid biosynthesis) and because different mechanisms may involve different fatty acid-microbe pairings and different environmental conditions.

### Lipids as Part of Epidermal Innate and Adaptive Immunity

The active role of epidermal lipids in the protection of the organism should be considered in the wider context of the innate immune response. The combination of environmental (acidity, humidity) and biochemical – either protein or lipid – factors of both host and microbial origin shapes the composition of the skin microbiota (Grice et al., 2009; Grice and Segre, 2011). All these components can directly interact with one another; for example, lipid and proteinaceous antimicrobial factors can have synergistic action, as in the case of sphingosine and LL-37 or sebaceous fatty acids and the antimicrobial histone H4 (Lee et al., 2009; Brogden et al., 2012).

In addition to their direct antimicrobial activity, fatty acids contribute to the acidification of the surface of the skin – the so-called “acid mantle” (Schade and Marchionini, 1928) – which in turn influences which micro-organisms can successfully colonize the skin and contributes to epidermal integrity (Puhvel et al., 1975; Fluhr et al., 2001; Hachem et al., 2003).

A fascinating example of the crosstalk between the immune system and the epidermal “shield” is the ability of epidermis-resident innate lymphoid cells to limit the growth of sebocytes and influence the presence of antimicrobial fatty acid in the sebum. To be retained in the tissue, innate lymphoid cells require secretion of chemokines and cytokines by the epithelium. If the lymphoid cells are lost, the epidermis reacts by producing more sebum and increasing sebaceous antimicrobial lipid content, in turn regulating the composition of the commensal microbiota (Kobayashi et al., 2019). In another instance that underscores the close relationship between the regulation of lipid production and immune response, overexpression of the transcription factor GATA6 in cultured sebocytes leads to alterations in the expression of lipid-modifying enzymes and reduced accumulation of lipids after stimulation with the PPARγ agonist troglitazone, while at the same time increasing the levels of anti-inflammatory mediators such as PD-L1 and IL10 (Oulès et al., 2020).

Some antimicrobial lipids have anti-inflammatory activity. Sapienic acid inhibits gene expression resulting from lipopolysaccharide stimulation in murine macrophages (Astudillo et al., 2018). Though a relatively high concentration of the fatty acid was necessary to see the effect (25 μM), it is still consistent with an anti-inflammatory action in skin, given the abundance of sapienic acid in sebum (>100 μM). Phytosphingosine demonstrates effective inhibition of interleukin-1α (IL-1α) secretion and PKC activation in skin explants and artificial human epidermis (Pavicic et al., 2007). Similar effects have been reported on a broader range of cytokines and chemokines (Klee et al., 2007; Brogden et al., 2012). In atopic dermatitis, a skin condition with an inflammatory component, levels of both sapienic acid and sphingosines are found to be downregulated (Arikawa et al., 2002; Takigawa et al., 2005).

**FIGURE 4:**
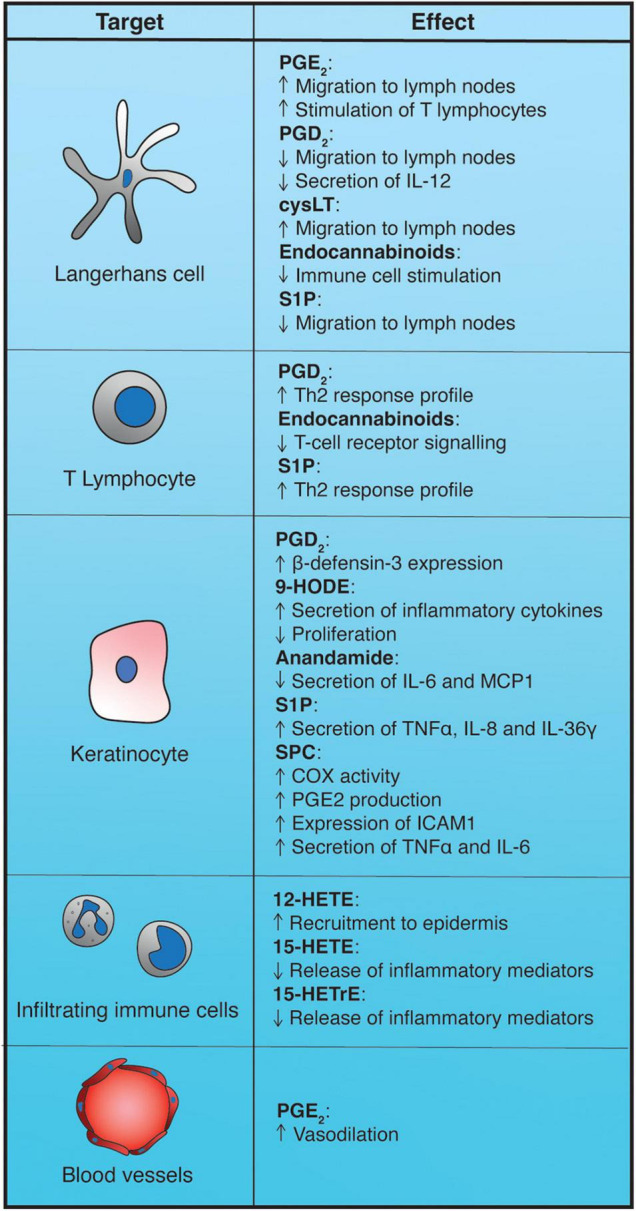
Impact of epidermal bioactive lipids on the immune response. 9-HODE, 9-hydroxyoctadecadienoic acid; 12-HETE, 12-hydroxyeicosatetraenoic acid; 15-HETrE, 15-hydroxyeicosatrienoic acid; cysLT, cysteinyl-leukotrienes; PGD_2_, prostaglandin D_2_; PGE_2_, prostaglandin E_2_; S1P, sphingosine-1-phosphate; SPC, sphingosylphosphorylcholine.

Lipids from numerous different classes and cellular origins can work as paracrine or autocrine mediators through cell-surface receptors to help determine the type of adaptive immune response that is triggered after an external stimulus perturbs the physiological equilibrium of the skin or to intervene in the regulation of epidermal cell behavior (Figure 4).

#### Eicosanoids and Related Lipids

Eicosanoids are a heterogeneous group of lipids derived from the metabolism of C20 poly-unsaturated fatty acids (PUFAs) such as arachidonic acid (C20:4ω6), dihomo-γ-linolenic acid (C20:3ω6), and eicosapentaenoic acid (C20:5ω3). These molecules, together with some related PUFAs, can be found in the epidermis and they can become substrates of a set of different enzymes. Cyclooxygenases can generate prostanoids (prostaglandins, prostacyclins, and thromboxanes). Lipoxygenases can give rise among others to leukotrienes, a variety of hydroxy-fatty acids [namely hydroxyeicosatrienoic acids (HETrEs), hydroxyeicosatetraenoic acids (HETEs), and hydroxyoctadecadienoic acids (HODEs)], and resolvins. In addition, cytochrome P450 enzymes lead to the production of such molecules as dihydro-eicosatetraenoic acids (DHETs) and epoxyeicosatetraenoic acid (EET). All these lipid species are known autocrine or paracrine biological mediators, with some of them being active in the epidermis (Kendall and Nicolaou, 2013).

Among prostanoids, prostaglandin E_2_ is one of the main mediators of inflammation in skin. PGE_2_ exerts its action by binding to four different G-protein-coupled receptors (GDPR), termed EP_1–4_ (Tober et al., 2007). In the context of normal epidermis, PGE_2_ has been mostly implicated as a vasodilation-promoting agent in the first phase of the sunburn response to acute UVB radiation. In human epidermis, levels of PGE_2_ increase between 6 and 24 h after UVB irradiation, leading to the development of erythema within 48 h (Black et al., 1978; Rhodes et al., 2001, 2009). In mice, it has been shown that PGE_2_ produced in the epidermis exerts its vasodilatory action by binding to EP_2_ and EP_4_ receptors on blood vessels (Kabashima et al., 2007). In addition to its effects following UVB irradiation, PGE_2_ possesses activity in Langerhans cells (epidermal dendritic cells). In a murine model, ablation of EP_4_ receptor impaired Langerhans cell migration toward the lymph nodes following antigen stimulation and the ability of Langerhans cells to stimulate T lymphocytes, suggesting a positive modulation of these processes by PGE_2_ (Kabashima et al., 2003).

PGD_2_ can be produced in the epidermis by Langerhans cells or by sub-epidermal mast cells (Lewis et al., 1982; Maciejewski-Lenoir et al., 2006). PGD_2_ can skew the profile of the T-cell response induced by antigen presentation toward Th2 cell activation by inhibiting the secretion of IL-12 from dendritic cells (Theiner et al., 2006). Accordingly, PGD_2_ promotes the recruitment and activation of mast cells and eosinophils characteristic of a Th2-type immune response. This effect is mediated by PGD_2_ binding to prostaglandin receptor DP_2_, also known as CRTH2, a GPCR that triggers inhibition of adenylate cyclase and lowers cAMP levels (G_α*i*_ signaling), ultimately leading to cellular mobilization (Hirai et al., 2001; Spik et al., 2005). PGD_2_ is able to induce expression of β-defensin-3 (hBD-3) in keratinocytes by engaging a src/MEK/ERK/c-Fos signaling axis, through DP_2_, and can thus bolster the antimicrobial defense of the epidermis. Moreover, since hBD-3 can trigger mast cell activation and further release of PGD_2_, this leads to the establishment of a positive feedback loop of communication between mast cells and keratinocytes (Kanda et al., 2010).

PGD_2_ can also be released by the helminth parasite *Schistosoma mansoni* to inhibit the migration of Langerhans cells to the lymph nodes following tumor necrosis factor-α (TNF-α) stimulation. In this case, the release of Langerhans cells is impaired by PGD_2_ binding to prostaglandin receptor DP_1_ and is dependent on the subsequent adenylate cyclase activation and rise in cAMP levels (G_α*s*_ signaling). Increased G_α*s*_ signaling is proposed to lead to cytoskeletal rearrangements and inhibition of cellular movement (Angeli et al., 2001, 2004).

While there is some evidence that resident epidermal cells express 5-lipoxygenase (5-LOX) – one of the key enzymes for the generation of leukotrienes – the major source of leukotriene production in the epidermis is thought to be the infiltrating immune cells (Janssen-Timmen et al., 1995; Breton et al., 1996). Leukotrienes, particularly cysteinyl-leukotrienes such as LTC_4_, have been implicated in the migration of Langerhans cells toward the lymph nodes, too, as mice lacking 5-LOX display a strongly reduced Langerhans cell movement (Robbiani et al., 2000; Doepping et al., 2007).

Epidermal cells can also express 12-LOX and 15-LOX. While 12-HETE is able to attract neutrophils and monocytes to the epidermis (Dowd et al., 1985), 15-LOX downstream products appear to have anti-inflammatory activity. 15-HETE and 15-HETrE can inhibit the release of inflammatory mediators such as leukotriene B4 (LTB_4_) from immune cells (Ziboh et al., 2000). Mice deficient for 15-LOX exhibit dramatic inflammatory skin phenotypes, including extensive immune infiltrates in the skin, epidermal hyperproliferation and compromised barrier function. These effects are at least partially due to a deficiency in resolvin D2 (Kim et al., 2018).

Oxidative stresses such as UVB radiation can cause oxidation of linoleic acid in the *stratum corneum* to the eicosanoid-related species 9-HODE. This mediator can bind the G2A receptor on keratinocytes, leading to the release of inflammatory cytokines and inhibition of proliferation through cell cycle arrest and DNA synthesis suppression. G2A is also induced by oxidative stress, making for a co-ordinated ligand/receptor response to an external insult (Hattori et al., 2008). This mechanism is particularly interesting, as it shows that an essential component of the epidermal barrier structure also functions as a sensor for damage to the barrier itself, highlighting how lipids can have multi-faceted functions within the epidermis.

#### Endocannabinoids

Endocannabinoids are endogenous lipids that can bind and activate GPC cannabinoid receptors (CB_1_ and CB_2_). The most prominent members of this class of compounds are arachidonic acid metabolites N-arachidonoyl-ethanolamine (anandamide) and 2-arachdonoylglycerol (2-AG). Anandamide can work as a partial agonist for CB_1_ and CB_2_ receptors, while 2-AG functions as a full ligand for both receptors (Sugiura et al., 2006; Smita et al., 2007). Biosynthesis of these compounds happens “on demand” in cell membranes through the action of N-acyltransferase and N-acyl-phosphatidylethanolamine phospholipase D in the case of anandamide, or the activity of phospholipase C and diacylglycerol lipase in the case of 2-AG (Wang and Ueda, 2009). The production of these two endocannabinoids occurs in epidermal keratinocytes and melanocytes, which also express both CB_1_ and CB_2_ as well as transient receptor potential vanilloid-1 (TRPV-1) which can also be activated by endocannabinoids (Maccarrone et al., 2003; Tóth et al., 2011; Pucci et al., 2012).

Generally, endocannabinoids possess anti-inflammatory activity, being able to suppress T cell receptor signaling and inhibit dendritic cell-mediated immune stimulation (Wacnik et al., 2008; Börner et al., 2009). In skin, the endocannabinoid system has been implicated in the attenuation of the inflammatory response during contact hypersensitivity reactions. When challenged with an obligate contact allergen, mice lacking cannabinoid receptors or treated with receptor antagonists show increased signs of inflammation and immune infiltration. Conversely, mice that accumulate anandamide due to the lack of its catabolic enzyme fatty acid amide hydrolase (FAAH) have reduced inflammatory responses after allergen treatment. Interestingly, stimulation by the contact allergen produces an increase in the levels of anandamide and 2-AG. Monocyte chemotactic protein 2 (MCP2) is one of the genes most upregulated in the absence of cannabinoid receptors (Karsak et al., 2007). Consistent with this, anandamide can lower the levels of IL-6 and MCP1 released by keratinocytes following TNF-α stimulation (Leonti et al., 2010).

#### Sphingosine-1-Phosphate

The hydrolysis of ceramides by ceramidases can release sphingosine bases that become substrates for sphingosine kinases, yielding sphingosine-1-phosphate (S1P). Due to its relatively low hydrophobicity and presence of a polar headgroup, S1P can exit the membrane space and move into solution but is not able to readily flip-flop across membrane leaflets. S1P is mostly found in serum and interstitial fluid, is strongly released during platelet degranulation, and exerts its actions in a paracrine or autocrine manner through GPCRs (termed S1P_1–5_) as well as intracellularly (Hannun and Obeid, 2008; van Meer et al., 2008; Gomez-Larrauri et al., 2020).

Sphingosine-1-phosphate generally behaves as a mitogen and a migration-stimulatory agent. However, some of its effects in skin go against this trend. S1P interferes with the migration of Langerhans cells toward the lymph node (Reines et al., 2009). Additionally, it affects how these dendritic cells stimulate T lymphocytes by promoting Th2 cell activation (Müller et al., 2005).

Sphingosine-1-phosphate is also involved in the response to bacterial invasion. Secretions from *P. aeruginosa* and *S. aureus* contain ceramidase or sphingomyelinase, respectively, that can stimulate the production of S1P. Subsequent binding to S1P_1_ and S1P_2_ receptors on the surface of keratinocytes can trigger the expression and release of inflammatory cytokines such as TNFα, IL-8, and IL-36γ, making S1P function as an “alarm signal” for the immune response (Oizumi et al., 2014; Igawa et al., 2019).

#### Sphingosylphosphorylcholine

Another sphingolipid, sphingosylphosphorylcholine (SPC, also known as lysosphingomyelin), arising from the action of sphingomyelin deacylase on sphingomyelin, has biological activity in epidermal cells. SPC is sufficiently polar to be able to move between different membranes and enter solution; it is thought to exert its effects through low-affinity binding to S1P receptors as well as intracellularly (Nixon et al., 2008; van Meer et al., 2008). SPC has pro-inflammatory effects on keratinocytes, increasing production of reactive oxygen species, activating COX-2 and increasing PGE_2_ production (Choi et al., 2010). Consistent with this inflammatory phenotype, SPC can also induce keratinocytes to express intercellular adhesion molecule-1 (ICAM-1), a surface protein necessary for leukocyte recruitment and retention, as well as the secretion of cytokines TNFα and IL-6. These phenotypes are in line with the remarkable increase in SPC levels seen in atopic dermatitis patients (Imokawa et al., 1999).

## Under-Recognized Actors – the Role of Lipid Signaling in Epidermal Homeostasis

For nearly a century of research, lipids have played a center-stage role in studies of the structural integrity and protection of the skin barrier. However, recent evidence is beginning to reveal an even more dynamic role for lipid mediators in signaling events that are key to the maintenance of epidermal homeostasis. Lipid molecules can act in an autocrine, paracrine or intracellular fashion to modulate several aspects of epidermal cell behavior, particularly keratinocyte differentiation, and the extent of their action has likely been underappreciated so far. In this respect, it is important to understand the enormous diversity of lipid species present in the epidermis and how this impacts the biology of the skin.

### Lipid Modulation of Epidermal Cell Behavior

The journey of a keratinocyte from basal layer stem cell to *stratum corneum* corneocyte is influenced by several cellular signaling pathways that control the balance between proliferation and differentiation, exit from the stem cell compartment and progression through the epidermal layers. Some of the prominent signaling molecules involved in these processes include β-catenin, extracellular-regulated kinase (ERK) and p38 mitogen-activated protein kinases (MAPK), phosphoinositide-3-kinase (PI3K)/RACα serine/threonine-protein kinase (Akt) and PKC. More specifically, accumulation of β-catenin in the nucleus promotes expansion of the stem cell compartment (Zhu and Watt, 1999; Watt and Collins, 2008) and high ERK activity is associated with more proliferative/stem-like keratinocytes (Hiratsuka et al., 2020). Conversely, p38 MAPK inhibition blocks the induction of differentiation of cultured human keratinocytes (Connelly et al., 2011). Activation of the PI3K/Akt pathway has been linked with the stimulation of keratinocyte differentiation (Janes et al., 2004, 2009). PKC is required for keratinocyte differentiation in response to a range of stimuli; for example blocking PKC can prevent cells from undergoing suspension-induced commitment and differentiation (Adhikary et al., 2010; Mishra et al., 2017).

**FIGURE 5:**
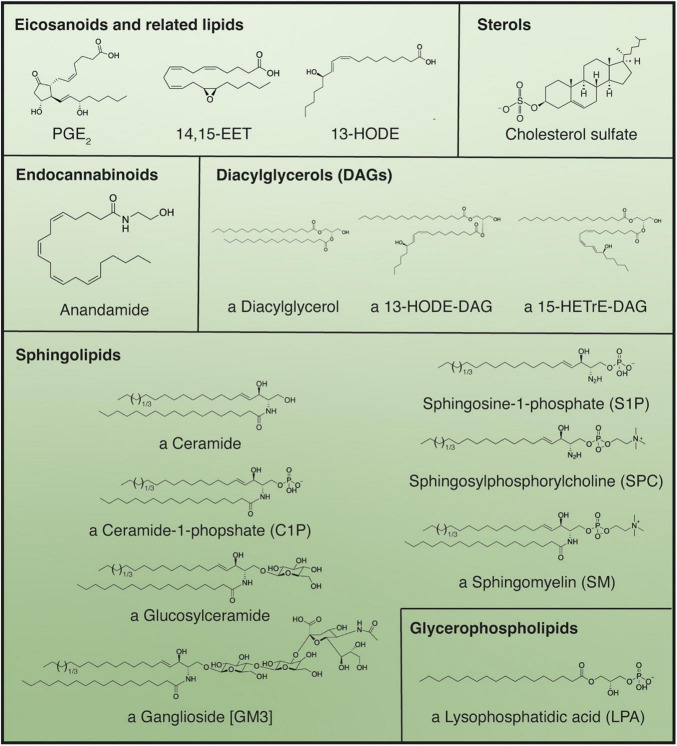
Main bioactive lipid species active in the regulation of keratinocyte differentiation. Use of the indeterminate article in front of compound name denotes if multiple structures for a certain compound are possible; in these cases, all “variable” fatty acid moieties are shown as C18:0 but can be substituted by alternative fatty acids differing in chain length and degree of unsaturation. Parentheses denote abbreviations, while brackets indicate the specific name of the structure displayed, when applicable.

Complementing our understanding of these well characterized signaling proteins, various different classes of lipids play a role in the regulation and maintenance of epidermal homeostasis (Figure 5).

#### Eicosanoids and Related Lipids

**FIGURE 6:**
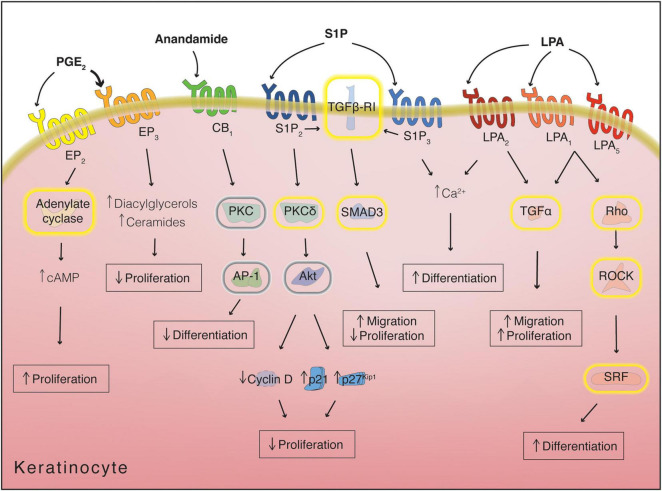
Receptor-mediated lipid signaling in keratinocytes. Yellow halos around proteins denote activation, while gray halos denote inhibition. Arrows next to metabolites/proteins denote increase or decrease in levels/expression. cAMP, cyclic adenosine monophosphate; LPA, lysophosphatidic acid; PGE_2_, prostaglandin E_2_; S1P, sphingosine-1-phosphate.

Besides their role in the modulation of the immune response, eicosanoids and related lipids also intervene in the regulation of keratinocyte differentiation. Epidermal keratinocytes express all four prostaglandin EP receptors bound by PGE_2_. The different receptors possess varying affinity for PGE_2_, with EP_1_ and EP_2_ having affinities in the nanomolar range, while EP_3_ and EP_4_ bind to PGE_2_ in the sub-nanomolar range. The lower-affinity EP_2_ receptor, expressed both basally and suprabasally, is responsible for stimulating keratinocyte proliferation, possibly in response to increased PGE_2_ levels during inflammatory events. This mitogenic effect is mediated by G_α*s*_ signaling and activation of adenylate cyclase (Konger et al., 1998, 2005a; Tober et al., 2007). In contrast, binding of PGE_2_ to the high affinity, basally expressed, EP_3_ receptor leads to keratinocyte growth inhibition, indicating a potential role for PGE_2_ in the limitation of basal cell proliferation in homeostatic conditions. Interestingly, EP_3_ receptor activation in this context does not affect adenylate cyclase activity but results in an increase in the levels of intracellular diacylglycerol and ceramide, both of which can promote keratinocyte differentiation. This suggests the tantalizing possibility of a lipid-mediated coupling mechanism between proliferation and differentiation in basal keratinocytes (Konger et al., 2005b; Figure 6).

The linoleic acid derivative 13-HODE can promote keratinocyte differentiation through activation of NF-κB (Ogawa et al., 2011). While the NF-kB pathway is mainly associated with inhibition of proliferation and protection form apoptosis in keratinocytes (Seitz et al., 2000), it was also shown to promote expression of differentiation-associated proteins such as Keratin 10 and Keratin 1. Treatment of keratinocytes with 13-HODE activates IκB kinase (IKK), which in turn phosphorylates and inactivates Inhibitor of κB (IκB) and stimulates NF-κB activity, leading to an increase in the expression of Keratin 1 (Ogawa et al., 2011).

**FIGURE 7:**
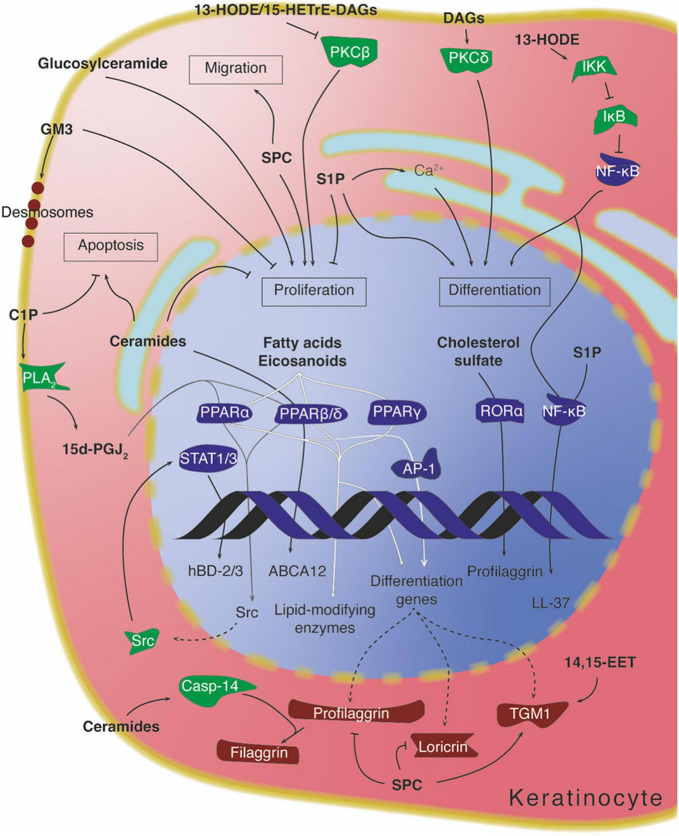
Intracellular lipid signaling in keratinocytes. Arrows denote activation, while “T” symbols denote inhibition. Arrows crossing the DNA symbol indicate transcription, while dotted lines represent translation. Arrows going through PPARs are depicted with different shadings for visual clarity. Protein colors indicate approximate function: transcription factors in dark blue, signaling mediators in green, and structural/differentiation-associated proteins in dark red. 13-HODE, 13-hydroxyoctadecadienoic acid; 14,15-EET, 14,15-epoxyeicosatrienoic acid; 15d-PGJ_2_, 15-deoxy-prostaglanding J_2_; 15-HETrE, 15-hydroxyeicosatrienoic acid; C1P, ceramide-1-phopshate; DAG, diacylglycerol; S1P, sphingosine-1-phosphate; SPC, sphingosylphosphorylcholine.

CYP2B19, expressed in the granular layer of mouse epidermis, produces 14,15-EET, which enhances the local activity of transglutaminases in the formation of the cornified cell envelope. The mechanisms through which this is achieved remain unclear, but no receptors for 14,15-EET have been robustly identified, suggesting that this mediator may be acting intracellularly on the complex regulation of transglutaminase activity (Figure 7; Gibson et al., 1996; Kalinin et al., 2002; Ladd et al., 2003).

#### Endocannabinoids

Anandamide inhibits keratinocyte differentiation through interaction with CB_1_, suppression of PKC and subsequent suppression of activator protein 1 (AP-1) transcription factor action (Figure 6). Keratinocytes treated with anandamide show decreased expression of cornified cell envelope proteins (such as involucrin and loricrin) and reduced transglutaminase activity. Keratinocyte anandamide levels are regulated during differentiation by increasing their degradation through FAAH (Maccarrone et al., 2003).

In melanocytes, endocannabinoids (likely from neighboring keratinocytes) can enhance melanin production by increasing tyrosinase expression following p38/ERK1/ERK2 and CREB activation downstream of CB_1_. Since keratinocytes can increase their anandamide synthesis in response to UVB radiation, the endocannabinoid system potentially represents a direct link between exposure to damaging sunlight and melanin production by melanocytes (Pucci et al., 2012).

#### Diacylglycerols and the Protein Kinase C Pathway

As mentioned above, the PKC pathway can be directly regulated by lipids. Depending on the isoform, PKC activity is modulated by both intracellular calcium levels and presence of diacylglycerol/lipids (-α -β -γ), by diacylglycerol/lipids alone (-δ, -ε, -η, -θ), or by allosteric interaction with other proteins (-ζ -ι/λ) (Nishizuka, 1992; Rosse et al., 2010). The two PKC isoforms that are most relevant to keratinocyte differentiation – PKCδ and PKCη – are insensitive to calcium but activated by the presence of phosphatidylserine and diacylglycerol (Adhikary et al., 2010). Moreover, lysophosphatidylcholines and unsaturated fatty acids can further enhance the activation of PKC (Bronfman et al., 1988; Shinomura et al., 1991; Nishizuka, 1992). Presence and activation of PKCδ and η, and the subsequent triggering of specific MAPK enzymes (MEKK, MEK-6, MEK-3, and p38δ), are necessary for the induction of keratinocyte differentiation in response to a number of stimuli (Figure 7; Adhikary et al., 2010; Mishra et al., 2017).

The specific acyl residues contained in diacylglycerol, as well as its hydrophilicity/hydrophobicity profile, can influence the degree to which PKC becomes regulated (Mori et al., 1982; Molleyres and Rando, 1988). In support of this, the eicosanoid-related molecule 13-HODE, a hydroxylated linoleic acid metabolite produced in the epidermis, becomes incorporated into membrane phosphatidylcholines and phosphatidylinositols, which in turn can be converted to 13-HODE-containing diacylglycerol (Cho and Ziboh, 1994b). This particular diacylglycerol is able to downmodulate the activity and expression of PKCβ (Figure 7). Interestingly, inhibition of this PKC isoform by 13-HODE has been correlated with the rescue of a keratinocyte hyperproliferation phenotype in guinea pigs (Cho and Ziboh, 1994a,^1995^; Ziboh et al., 2000). Similar findings were also reported for diacylglycerol containing the anti-inflammatory eicosanoid-related lipid 15-HETrE (Cho and Ziboh, 1997).

#### Sphingolipids

Sphingolipids are one of the most functionally multifaceted lipid classes in the epidermis. They play a fundamental role in the formation and the structure of the epidermal barrier and also participate in the protection of the epidermis. Additionally, several sphingolipid species take part in epidermal signaling (Hannun and Obeid, 2008; Gomez-Larrauri et al., 2020). These include ceramides, glycosylceramides, ceramides-1-phosphate, sphingosine-1-phosphate, and sphingosylphosphorylcholine. The metabolism of sphingolipids is remarkably interconnected, with interconversion between different species occurring readily depending on the relevant enzymes’ presence and abundance (Hannun, 1994; Hannun et al., 2001; Hannun and Obeid, 2008).

Ceramides can be formed *de novo* by the condensation of palmitate and serine catalyzed by serine palmitoyl transferase and the subsequent activity of ceramide synthase. Alternatively, they can be derived from either sphingomyelin through the action of sphingomyelinases or glucosylceramides through the action of glucocerebrosidase. They can be mainly found in the ER membranes (where their *de novo* synthesis occurs) or at the plasma membrane. Due to their physico-chemical properties they are unable to transfer freely between different cellular compartments, needing either vesicular transport or protein mediators to move across membranes. They can, however, flip-flop between membrane leaflets, though this may too be subject to regulation (Hannun and Obeid, 2008; van Meer et al., 2008). Ceramides act as signaling molecules in a variety of settings and are mostly known for inducing apoptosis and responding to cellular stress (Uchida, 2014; Uchida and Park, 2021). *In vitro*, treatment of keratinocytes with exogenous short-chained (C2–C8) ceramides can inhibit their proliferation and induce differentiation (Wakita et al., 1994).

Both exogenous short-chained (C2 and C6) ceramides and endogenous ceramides can directly regulate the expression of certain differentiation-related genes in keratinocytes. One example is glucosylceramide transporter ABCA12, involved in the translocation of glucosylceramides into lamellar bodies, whose transcription is enhanced by ceramide through increased PPARβ/δ expression (Jiang et al., 2009). Another case is the expression of caspase-14 – a protein present in differentiated keratinocytes and responsible for the processing of profilaggrin into filaggrin, important for the appropriate hydration of the *stratum corneum*. Caspase-14 expression can be directly stimulated by ceramides, although the mechanism does not involve PPARβ/δ (Jiang et al., 2013). C2 ceramide administered *in vitro* has been shown to strongly reduce Akt activity in melanocytes, to inhibit their growth, and to decrease tyrosinase activity and consequent melanin production (Kim et al., 2001). Ceramides can also intervene in the response of epidermal keratinocytes to UVB radiation. Exposing keratinocytes to UVB increases the *de novo* synthesis of ceramides, which in turn participate in the subsequent induction of apoptosis by a caspase-independent mechanism (Uchida et al., 2003; Figure 7).

Interfering with sphingolipid metabolism *in vivo* not only, as predicted, leads to disruption of epidermal homeostasis but has also a surprising impact on keratinocyte differentiation. Eliminating alkaline ceramidase, an enzyme responsible for ceramide degradation, in mouse skin leads to an abnormal accumulation of ceramides, which in turn compromise the barrier function of the epidermis and cause alterations in the final stages of keratinocyte differentiation and an increase in the epidermal stem cell population of the hair follicle (Liakath-Ali et al., 2016).

In apparent contrast, knockout of ceramide synthase 4 (CerS4), an enzyme responsible for ceramide synthesis, in mice produces an expansion of the hair follicle stem cell compartment (Peters et al., 2015). The different effects of alkaline ceramidase and CerS4 deletion can be reconciled by the fact that while the absence of alkaline ceramidase produces a generalized increase of all ceramide species, lack of CerS4 produces a decrease of some ceramide species, but an increase in others, indicating that only specific ceramide subspecies might influence the behavior of murine epidermal stem cells. In addition to its effect on the stem cell compartment, epidermal deletion of CerS4 impairs maintenance of the epidermal barrier in adult mice, leading to increased trans-epidermal water loss, hyperkeratosis and accumulation of immune cells. This effect is notable because CerS4 catalyzes the synthesis of shorter-chained ceramide species that are not directly involved in the structure of the barrier. These species or other lipids indirectly affected by CerS4 knockdown may thus be involved in signaling events that are necessary for epidermal homeostasis (Peters et al., 2020).

In contrast to ceramides, glucosylceramide has mitogenic effects on epidermal keratinocytes when administered subcutaneously in mice (Figure 7; Marsh et al., 1995). Conversely, certain glycosylceramides such as the ganglioside GM3, which are present on the outer leaflet of keratinocyte plasma membranes, have an antiproliferative effect when supplemented in the medium of cultured keratinocytes (Paller et al., 1993). Gangliosides (ceramides conjugated to oligosaccharides containing sialic acid) have been implicated in some important membrane dynamics in the early phases of keratinocyte differentiation. When basal keratinocytes commit to differentiation, the adherens junctions connecting the basal cells to one another must relax to allow the cells to delaminate to the suprabasal layers. In order for this process to occur, desmosomes containing the differentiation-dependent cadherin desmoglein 1 (Dsg1) must be localized within insoluble membrane compartments enriched in gangliosides. Interfering with the compartmentalization of Dsg1 in these lipid domains impairs the release of tension at the adherens junctions and leads to reduced keratinocyte delamination from monolayers (Figure 7; Nekrasova et al., 2018).

Ceramides-1-phosphate (C1Ps) are sphingolipid mediators that generally exert opposing effects to those of ceramides (e.g., increased cell survival, increased proliferation), and phosphorylation by ceramide kinase is usually considered a way in which ceramides can be inactivated. C1Ps can be found in the Golgi apparatus and the plasma membrane. They are limited in their mobility between membranes, and owing to their polar head group are unlikely to flip-flop between membrane leaflets (Hannun and Obeid, 2008; Uchida, 2014; Gomez-Larrauri et al., 2020). In keratinocytes, a role for C1P has been described during cellular stress. Serum starvation of mouse keratinocytes induces apoptosis which is mitigated by the activity of PPARβ/δ. Among its targets is ceramide kinase, whose activity is necessary to reduce the levels of intracellular ceramides and prevent excessive apoptosis (Tsuji et al., 2008). C1P produced in response to endoplasmic reticulum stress leads to an increase in the expression of β-defensin 2 and 3 (hBD-2/3). This suggests a model whereby C1P activates cellular phospholipase A_2_, causing the release of arachidonic acid from phospholipids and an increase of 15-deoxy-PGJ_2_; this leads to activation of PPARα or -β/δ, which promotes expression of Src and stimulates STAT1/3-mediated expression of the β-defensin genes (Figure 7; Kim et al., 2014).

Along with its function in immunity, spiingosine-1-phosphate (S1P) also inhibits proliferation and promotes differentiation of keratinocytes (Vogler et al., 2003). Binding of S1P to S1P_2_ antagonizes the proliferative signals coming from the insulin receptors by activating PKCδ, causing dephosphorylation of Akt and leading to inhibition of cyclin D2 and upregulation of cyclin-dependent kinase inhibitors p21 and p27*^Kip1^* (Kim et al., 2004; Schüppel et al., 2008). Moreover, signals mediated through S1P_3_ are reported to increase the intracellular calcium concentration in keratinocytes, which has been linked to differentiation (Watt et al., 1984; Lichte et al., 2008; Figure 6).

In addition to proliferation inhibition and in sharp contrast to its role in Langerhans cells, S1P stimulates keratinocyte migration. This effect is enacted by S1P receptors through transforming growth factor-β (TGF-β)-receptor I-mediated phosphorylation and activation of Smad3 (Figure 6). In fact, S1P and TGF-β treatment cause similar phenotypes in keratinocytes, both in terms of general cellular behavior and in terms of specific gene expression changes; for example, both ligands can increase laminin 5 production (Amano et al., 2004; Sauer et al., 2004). The overlap between S1P and TGF-β signaling is likely important during the complex orchestration of the wound healing process, when both these ligands as well as multiple other mediators are abundantly produced (Vogler et al., 2003; Rognoni and Watt, 2018).

Besides its activity through GPCRs, S1P can act intracellularly to influence the behavior of keratinocytes. Activation of sphingosine kinase 1, one of the two enzymes catalyzing S1P biosynthesis, mobilizes calcium from intracellular stores, leading to inhibition of keratinocyte proliferation and stimulation of differentiation (Hong et al., 2008). Similarly, inhibition of S1P lyase, the main enzyme responsible for S1P catabolism, increases intracellular S1P levels and causes cell cycle arrest and expression of differentiation markers (Jeon et al., 2020). In response to ER-stress, which can be caused for example by perturbation of the epidermal barrier, S1P levels rise and promote NF-κB activation *via* assembly of an intracellular signaling complex, causing the stimulation of C/EBPα-dependent transcription, and ultimately leading to increased expression of the cathelicidin LL-37 and enhanced epidermal protection (Park et al., 2013, 2016; Figure 7). The effect of ER-stress-induced S1P on cathelicidin LL-37 mimics that of E R-stress-induced C1P on β-defensins (Kim et al., 2014), resulting in a synergistic response to external injury *via* two related sphingolipid metabolites.

Sphingosylphosphorylcholine (SPC) appears to promote an abnormal route of differentiation, with increased intracellular calcium levels and activation of transglutaminase 1, at the same time as inhibiting the expression of cornified cell envelope proteins such as profilaggrin and loricrin (Higuchi et al., 2001; Choi et al., 2010). SPC also promotes re-epithelialization during wound healing by acting as a keratinocyte mitogen and increasing migration through stimulation of the expression of urokinase-type plasminogen activator (uPA) and its receptor (uPA-R) (Figure 7; Wakita et al., 1998).

Sphingomyelins (SMs) are synthesized in the Golgi apparatus and found at the plasma membrane. Their double aliphatic chain makes them insoluble in water and their polar phosphocholine group impairs flip-flopping between membrane leaflets (Hannun and Obeid, 2008; van Meer et al., 2008). Unlike other sphingolipids, SMs are a major component of cellular membranes, which makes them less likely to be able to act as bioactive mediators. In fact, SMs are often considered a reservoir for the generation of bioactive sphingolipids (Hannun, 1994; Hannun et al., 2001; Wanner et al., 2004). However, variation in SM levels can influence the biophysical characteristics of the membrane such as, for example, fluidity, stiffness and mechanosensory properties, which in turn can drive epidermal cellular responses (Mobasseri et al., 2019). Moreover, by virtue of the mostly saturated nature of its acyl chains, SMs can form lipid aggregates with cholesterol within membranes which can influence protein interaction dynamics. While the existence of such lipid “rafts” in biological membranes *in vivo* has been controversial, they have been observed in artificial bilayers mimicking the lipid composition of the plasma membrane (Dietrich et al., 2001; Samsonov et al., 2001; Simons and Sampaio, 2011; Levental et al., 2020). Early studies showed that epidermal desmosome preparations are enriched in cholesterol, SM and gangliosides (Drochmans et al., 1978); more recently, rafts have been implicated in desmosome assembly and dismantling in keratinocytes (Stahley et al., 2014).

#### Lysophosphatidic Acid

Lysophosphatidic acids (LPAs) are the simplest phospholipid, formed by a glycerol backbone attached to a phosphate group, a fatty acid, and a hydroxyl moiety. LPA can be synthesized intracellularly by glycerophosphate acyltransferase, acylglycerol kinase or phospholipase A. Alternatively, it can be generated extracellularly by the secreted lysophospholipase D autotaxin. Degradation of LPA is mainly carried out by lipid phosphate phosphatases. The single aliphatic chain of LPA makes it sufficiently soluble to leave membranes (van Meer et al., 2008; Lei et al., 2018).

In addition to being a precursor in phospholipid biosynthesis, LPA is a ligand of a series of GPCRs – LPA_1–6_ – through which it exerts its biological activity. Some of these receptors belong to the same family as S1P receptors, and S1P and LPA share some structural similarity (Geraldo et al., 2021). A dependence of the biological activity of LPA on its acyl-chain length has been reported (van Corven et al., 1992).

During wound repair, LPA is delivered to the injury site mainly through platelet degranulation and its effects are akin to S1P: keratinocyte migration is enhanced, with increases in expression of laminin-332, TGF-α and TGF-β. Unlike S1P, however, LPA appears to stimulate keratinocyte proliferation, although this may be an indirect consequence of the induction of TGF-α expression rather than direct LPA-mediated signaling (Figure 6; Piazza et al., 1995; Demoyer et al., 2000; Amano et al., 2004). An interesting study examining skin blisters showed that LPA production is increased in blister fluid by autotaxin activity and expression of LPA1 is also enhanced, pointing to the relevance of this signaling *in vivo* (Mazereeuw-Hautier et al., 2005).

When treating keratinocytes with LPA, an induction of differentiation can be observed, potentially through multiple pathways. LPA induces an increase of intracellular calcium concentration, likely through binding to LPA_2_ (Lichte et al., 2008). Binding to LPA_1_ or LPA_5_ instead leads to an increase in the differentiation marker pro-filaggrin and alteration of cellular morphology, signaling *via* G_α12/13_ to RhoA-ROCK and activating Serum Response Factor (SRF) (Figure 6). The SRF pathway is involved in keratinocyte differentiation induced by restricted spreading on micropatterned substrates (Connelly et al., 2010; Sumitomo et al., 2019). The increase in filaggrin triggered by LPA treatment provides a likely molecular explanation for the beneficial effects of LPA on skin moisturization (Yahagi et al., 2011).

#### Peroxisome Proliferator-Activated Receptors Signaling

Other lipid-sensitive proteins that have been shown to be involved in the regulation of keratinocyte differentiation are peroxisome proliferator-activated receptors (PPARs) and liver X receptors (LXRs). When activated by a lipid ligand, PPARs or LXR heterodimerize with retinoid X receptor (RXR) and regulate gene expression. Three PPAR isoforms (α-, β/δ-, γ-) exist, all of which are expressed in keratinocytes: while PPARβ/δ expression is constitutive, PPARα and PPARγ levels increase with differentiation (Rivier et al., 1998). Both LXR isoforms – α and β – are constitutively expressed in human keratinocytes (Russell et al., 2007). PPAR endogenous ligands are fatty acids and their metabolites, including eicosanoids, while LXRs are activated by the cholesterol metabolite oxysterol (Bocos et al., 1995; Janowski et al., 1996).

Activation of all PPAR isoforms or LXRs pushes keratinocytes to differentiate both *in vitro* in human keratinocytes and *in vivo* in mouse epidermis (Hanley et al., 1998, 2000b,a; Mao-Qiang et al., 2004; Schmuth et al., 2004). More specifically, while all receptors stimulate expression of differentiation markers, PPARα and LXR also cause inhibition of keratinocyte proliferation (Hanley et al., 1998, 2000b). PPARs or LXR activation induce accumulation of lipids as well as expression of lipid metabolism enzymes, including ones involved in lamellar body biogenesis, and stimulate lamellar body secretion when activated topically in mouse epidermis (Rivier et al., 2000; Man et al., 2006; Jiang et al., 2008, 2010; Lu et al., 2010). While the expression of lipid-modifying enzymes appears to be directly regulated by the receptors’ transcriptional activity, the kinetics of induction of differentiation markers suggests an indirect regulation (Hanley et al., 1998; Schmuth et al., 2004).

In the case of PPARα and LXL, differentiation proceeds through the activation of AP-1 transcription factors (Hanley et al., 2000a,b). While there is overlap between the consequences of activating different PPAR isoforms, the different isoforms are able to exert their effect independently of one another, which suggests a certain degree of redundancy. The fact that PPARs and LXRs are sensitive to endogenous lipid mediators generated during keratinocyte differentiation and can affect the transcription of differentiation proteins has been proposed as a coupling mechanism to coordinate the changes in lipids and proteins occurring during epidermal maturation (Figure 7; Rivier et al., 2000; Man et al., 2006; Jiang et al., 2008, 2010; Lu et al., 2010).

#### Cholesterol Sulfate

Cholesterol metabolites can also participate in the modulation of keratinocyte differentiation. An example of this is cholesterol sulfate, whose levels progressively increase over the course of differentiation, peaking in the granular layer. Treatment of human keratinocytes with cholesterol sulfate causes induction of the expression of the retinoic acid receptor-related orphan receptor α (RORα), which in turn is able to elicit transcription of the profilaggrin gene (Hanyu et al., 2012; Figure 7). This mechanism provides another link between the constantly changing protein and lipid landscape of differentiating keratinocytes, further underscoring the functional connection of these changes in the creation of the epidermal barrier.

### An Unbiased Screen for Lipid Regulators of Differentiation

While we have presented evidence that separate lipid classes play a role in signaling to impact keratinocyte proliferation, differentiation and other processes, this is inevitably an oversimplification. The lipid species produced in the cell and the enzymes that modify them form an extremely intricate network and it is difficult to predict the multiple perturbations that ensue from a specific intervention, such as depletion of a single lipid modifying enzyme (Muro et al., 2014). Lipid subspecies can influence cellular biology in ways that go beyond their direct interaction with proteins as ligands or allosteric modulators, by for example working as ensembles that can facilitate (or impede) the activity of groups of molecular partners (Muro et al., 2014).

Despite these challenges, newly developed techniques or refinement of established methods are beginning to shed more light on the potential contribution of endogenous cellular lipid subspecies to a variety of cellular processes. Prompted by the finding that the alteration of the ceramide profile in mouse epidermis could lead to an effect on the stem cell compartment (Peters et al., 2015; Liakath-Ali et al., 2016), our lab recently performed lipidomics in a well characterized *in vitro* keratinocyte differentiation system combined with a lipid-modifying enzyme siRNA screen to evaluate the changes in cellular lipid composition occurring during differentiation and to try to understand which changes might be important for the early phases of this process.

Our study identified several ceramide and glucosylceramide subspecies that could induce keratinocyte differentiation when administered exogenously *in vitro* (Vietri Rudan et al., 2020). Using such a combined approach it was possible to leverage the high specificity of protein-targeting techniques while keeping the focus on the lipid changes. This is key because of the cascading effects of lipid enzyme perturbation and the potential presence of compensatory mechanisms. In line with this, while the enzymes that emerged from our siRNA screen (ELOVL1 and FATP1) were directly associated with generation of specific chain-length saturated and mono-unsaturated fatty acids and fatty acyl CoAs, the bioactive lipids identified belonged to various different phospholipid and sphingolipid classes.

While our approach demonstrates the power of an unbiased lipidomic screen, it is important to note that our lipidomic panel did not include a number of potentially relevant lipid classes. For example, we did not include fatty acids and fatty acyl-CoAs, which may regulate keratinocyte differentiation through, for example, PPAR or PKC signaling (Bronfman et al., 1988; Bocos et al., 1995). In addition, the size of the effect of bioactive lipids we observed experimentally will depend as how efficiently they reach the appropriate subcellular destination. Furthermore, lipids operate by affecting the functionality of whole assemblies of molecular partners, including proteins and carbohydrates, which makes it harder to evaluate their role using the linear approaches that are typically employed when studying protein signaling pathways.

## Discussion

We have described how in the epidermis the physical, chemical and biological characteristics of lipids are all functionally leveraged to build, protect, and maintain the outer barrier of the organism. The hydrophobic and fluidity properties of ceramides, cholesterol and fatty acids are exploited to create an impermeable, resistant, yet flexible, dynamic structure in the *stratum corneum*. Nevertheless, despite nearly one hundred years of research into the physiology of the body’s outer boundary, questions still remain as to how exactly different ceramide species participate in the generation of the exquisite biophysical properties of the cornified layer. This is not surprising, considering that the composition of the *stratum corneum* can vary quite extensively in different species (e.g., in birds), while maintaining its fundamental microstructure and functional characteristics (Champagne et al., 2012). Similarly, mammals living in cold environments, such as arctic foxes or reindeer, have dramatically lower melting points for the lipid mixtures isolated from their extremities as compared to the ones obtained from the body core, reflecting the fact that their peripheral tissues can be up to 40°C colder than their central tissues (Irving et al., 1957). How this adaptation can influence the properties, microstructure, and composition of the epidermis of these animals is yet to be fully elucidated.

Far from being only passive building blocks, lipids also protect the epidermis by way of their chemical features, as in the fatty acid contribution to the “acid mantle,” and biological activity against microbes, significantly contributing to shape the makeup of the commensal microbiota. The crosstalk between skin surface resident microbes and host factors is extensive. Numerous lines of research have shown that the normal skin microflora can modulate the expression of antimicrobial peptides to protect from opportunistic pathogens (Gallo and Nakatsuji, 2011). Lipases of microbial origin are mainly responsible for the presence of fatty acids in the sebum, and disruption of the normal microbiome can lead to altered sebum composition. Conversely, the relative abundance of sebaceous secretions can determine the abundance of different bacterial species at specific body sites (Table 1; Grice et al., 2009; Grice and Segre, 2011). Some parasites can secrete lipids to interfere with the host’s immune response, as is the case for PGD_2_ secreted by *S. Mansoni* (Angeli et al., 2001). The full range of lipid mediators of microbial origin that participate in their interaction with the host remains to be explored.

The participation of secreted lipid species of keratinocyte or sebaceous origin n innate immunity provides a fascinating insight into how different systems in the body can act in unison to ensure the preservation of homeostasis and the appropriate response to external perturbations, and how even foreign micro-organism colonization can be used as a means to maintain and protect the outer limit of the body (Gallo and Nakatsuji, 2011; Kobayashi et al., 2019). Gaining a fuller picture of how antimicrobial lipids act on their targets, intervene in the inflammatory response and communicate with the innate and adaptive immune system may help us in the development of new, more effective, treatments against pathogens or inflammatory disorders.

With lipids playing such an important functional role in the epidermis, it is not surprising that they intervene in the regulation of keratinocyte differentiation and in the maintenance of epidermal homeostasis. In this review, we have highlighted numerous examples of how bioactive lipid mediators from several different classes can affect in these processes, whether by influencing membrane characteristics or participating in cellular signaling. The importance of membrane properties in keratinocytes is highlighted for example by the interaction between key proteins involved in their differentiation, desmosomal cadherins, and membrane lipid domains to ensure the appropriate assembly and disassembly of these functional ultrastructures and in turn guarantee proper delamination from the basal layer (Stahley et al., 2014; Nekrasova et al., 2018).

More generally, the apico-basal polarity of the plasma membrane is a fundamental feature of epithelial cells, with keratinocytes being no exception; the relevance of lipids in the establishment of such oriented cell membranes remains poorly understood and represents a major avenue for future research (Ikenouchi, 2018). Moreover, lipids can influence membrane stiffness, fluidity and mechanosensitivity, which are known to have an effect on keratinocyte behavior (Mobasseri et al., 2019). However, the extent to which lipids may contribute to the regulation of these membrane properties, the mechanisms and species involved, still needs to be fully characterized.

There are many factors that can contribute to the biological activity of lipids. While variation in levels can be a good indication of the involvement in a biological process – as for example in the case of cholesterol sulfate in the *stratum corneum* (Elias et al., 1984; Sato et al., 1998; Hanyu et al., 2012) – there are other layers of regulation that likely influence lipid-mediated regulation of cellular behavior. A characteristic of several lipid classes is their limited ability, owing to physical and chemical constraints, to move between different cellular environments or even across the two leaflets of the same membrane (Hannun and Obeid, 2008; van Meer et al., 2008). Therefore, cells can selectively target the activity of specific lipid players to certain subcellular locales by modulating the expression and activation of transport molecules and vesicular trafficking processes. The importance of this level of regulation in the epidermis is underscored by the diversity of lipid transporters expressed in keratinocytes and the emergence of severe pathological conditions associated with transporter loss or dysfunction (Elias et al., 2008; Khnykin et al., 2011; Vietri Rudan et al., 2020).

In the case of the modulation of keratinocyte differentiation, it often remains unclear to what degree the activity of endogenously generated individual lipid mediators is important and at what stage of the *in vivo* differentiation process they might come into play. An example of this is the fatty acid-mediated regulation of PPARs: while it is established that fatty acids can regulate PPAR activity and that they are abundant in keratinocytes, it is still not clear whether they do so endogenously, at what stage of the differentiation process, what PPAR isoforms are involved and, importantly, what fatty acids. This last question points toward another key fact to be considered: the cell produces a vast amount of lipid subspecies (varying for example in carbon chain length, number, and location of unsaturations, and hydroxylation profile) whose influence on cellular behavior is still poorly understood and has only recently begun to be investigated. A combination of lipid enzyme targeting and lipidomics has identified individual lipid subspecies from multiple classes that can influence keratinocyte differentiation *in vitro*, underscoring the still little-explored regulatory potential of the cellular lipid repertoire (Vietri Rudan et al., 2020). Such comprehensive strategies can pinpoint individual lipid actors that can take part in signaling events or provide indications as to what lipid properties matter most within a certain process. Indeed, studies are beginning to shed light on the influence of this diverse collection of lipid molecules on several cellular and biological activities, such as cell division or immune cell activation (Atilla-Gokcumen et al., 2014; Köberlin et al., 2015).

Challenges still remain to explore some of the more elusive aspects of lipid biology, for example how the cellular distribution and/or traffic of bioactive lipids among the various membranous organelles may play into their functionality, or how much individual lipid species or lipid features matter for epidermal homeostasis *in vivo*. Technological advances will be key to answering these complex questions. In this respect, implementation of recently developed, sophisticated techniques, like single-cell lipidomics (Capolupo et al., 2021; Li et al., 2021), already promises more exciting developments in unraveling the finer details of the roles played by lipids in the regulation of cellular behavior (Züllig et al., 2020; Capolupo et al., 2021; Li et al., 2021). We are confident that in the future the application of these new technologies will provide a clearer picture of the rich and essential contribution of lipid molecules to epidermal biology.

## Author Contributions

MV and FW wrote and edited the text. MV designed the figures. Both authors contributed to the article and approved the submitted version.

## Conflict of Interest

The authors declare that the research was conducted in the absence of any commercial or financial relationships that could be construed as a potential conflict of interest.

## Publisher’s Note

All claims expressed in this article are solely those of the authors and do not necessarily represent those of their affiliated organizations, or those of the publisher, the editors and the reviewers. Any product that may be evaluated in this article, or claim that may be made by its manufacturer, is not guaranteed or endorsed by the publisher.

## References

[B1] Adhikary G. Chew Y. C. Reece E. A. Eckert R. L. (2010). PKC-delta and -eta, MEKK-1, MEK-6, MEK-3, and p38-delta are essential mediators of the response of normal human epidermal keratinocytes to differentiating agents. *J. Invest. Dermatol.* 130 2017–2030. 10.1038/jid.2010.108 20445555 PMC3120227

[B2] Akiyama M. (2017). Corneocyte lipid envelope (CLE), the key structure for skin barrier function and ichthyosis pathogenesis. *J. Dermatol. Sci.* 88 3–9. 10.1016/j.jdermsci.2017.06.002 28623042

[B3] Aly R. Maibach H. I. Rahman R. Shinefield H. R. Mandel A. D. (1975). Correlation of human in vivo and in vitro cutaneous antimicrobial factors. *J. Infect. Dis.* 131 579–583. 10.1093/infdis/131.5.579 805186

[B4] Aly R. Maibach H. I. Shinefield H. R. Strauss W. G. (1972). Survival of pathogenic microorganisms on human skin. *J. Invest. Dermatol.* 58 205–210. 10.1111/1523-1747.ep12539912 4623202

[B5] Amano S. Akutsu N. Ogura Y. Nishiyama T. (2004). Increase of laminin 5 synthesis in human keratinocytes by acute wound fluid, inflammatory cytokines and growth factors, and lysophospholipids. *Br. J. Dermatol.* 151 961–970. 10.1111/j.1365-2133.2004.06175.x 15541073

[B6] Angeli V. Faveeuw C. Roye O. Fontaine J. Teissier E. Capron A. (2001). Role of the parasite-derived prostaglandin D2 in the inhibition of epidermal langerhans cell migration during schistosomiasis infection. *J. Exp. Med.* 193 1135–1148. 10.1084/jem.193.10.1135 11369785 PMC2193325

[B7] Angeli V. Staumont D. Charbonnier A.-S. Hammad H. Gosset P. Pichavant M. (2004). Activation of the D Prostanoid receptor 1 regulates immune and skin allergic responses. *J. Immunol.* 172 3822–3829. 10.4049/jimmunol.172.6.3822 15004188

[B8] Arikawa J. Ishibashi M. Kawashima M. Takagi Y. Ichikawa Y. Imokawa G. (2002). Decreased levels of sphingosine, a natural antimicrobial agent, may be associated with vulnerability of the stratum corneum from patients with atopic dermatitis to colonization by *Staphylococcus aureus*. *J. Invest. Dermatol.* 119 433–439. 10.1046/j.1523-1747.2002.01846.x 12190867

[B9] Astudillo A. M. Meana C. Guijas C. Pereira L. Lebrero P. Balboa M. A. (2018). Occurrence and biological activity of palmitoleic acid isomers in phagocytic cells. *J. Lipid Res.* 59 237–249. 10.1194/jlr.m079145 29167413 PMC5794419

[B10] Atilla-Gokcumen G. E. Muro E. Relat-Goberna J. Sasse S. Bedigian A. Coughlin M. L. (2014). Dividing cells regulate their lipid composition and localization. *Cell* 156 428–439. 10.1016/j.cell.2013.12.015 24462247 PMC3909459

[B11] Beck S. Sehl C. Voortmann S. Verhasselt H. L. Edwards M. J. Buer J. (2020). Sphingosine is able to prevent and eliminate *Staphylococcus epidermidis* biofilm formation on different orthopedic implant materials in vitro. *J. Mol. Med.* 98 209–219. 10.1007/s00109-019-01858-x 31863153 PMC7007894

[B12] Beddoes C. M. Rensen D. E. Gooris G. S. Malfois M. Bouwstra J. A. (2021). The importance of free fatty chain length on the lipid organization in the long periodicity phase. *Int. J. Mol. Sci.* 22:3679. 10.3390/ijms22073679 33916267 PMC8038103

[B13] Behne M. Uchida Y. Seki T. de Montellano P. O. Elias P. M. Holleran W. M. (2000). Omega-Hydroxyceramides are required for Corneocyte Lipid Envelope (CLE) formation and normal epidermal permeability barrier function. *J. Invest. Dermatol.* 114 185–192. 10.1046/j.1523-1747.2000.00846.x 10620136

[B14] Bergsson G. Arfinsson J. Steingrímsson Ó Thormar H. (2001). Killing of Gram-positive cocci by fatty acids and monoglyceridesNote. *APMIS* 109 670–678. 10.1034/j.1600-0463.2001.d01-131.x 11890570

[B15] Bibel D. J. Aly R. Shinefield H. R. (1992). Antimicrobial activity of sphingosines. *J. Invest. Dermatol.* 98 269–273. 10.1111/1523-1747.ep12497842 1545135

[B16] Bibel D. J. Aly R. Shah S. Shinefield H. R. (1993). Sphingosines: antimicrobial barriers of the skin. *Acta Derm-venereol.* 73 407–411. 7906449 10.2340/0001555573407411

[B17] Black A. Greaves M. Hensby C. Plummer N. (1978). Increased prostaglandins E2 and F2alpha in human skin at 6 and 24 h after ultraviolet B irradiation (290-320 nm). *Br. J. Clin. Pharmacol.* 5 431–436. 10.1111/j.1365-2125.1978.tb01650.x 656282 PMC1429345

[B18] Bocos C. Göttlicher M. Gearing K. Banner C. Enmark E. Teboul M. (1995). Fatty acid activation of peroxisome proliferator-activated receptor (PPAR). *J. Steroid Biochem. Mol. Biol.* 53 467–473. 10.1016/0960-0760(95)00093-f 7626496

[B19] Börner C. Smida M. Höllt V. Schraven B. Kraus J. (2009). Cannabinoid receptor Type 1- and 2-mediated increase in cyclic AMP inhibits T Cell receptor-triggered signaling*. *J. Biol. Chem.* 284 35450–35460. 10.1074/jbc.m109.006338 19858202 PMC2790974

[B20] Borst P. Loos J. A. Christ E. J. Slater E. C. (1962). Uncoupling activity of long-chian fatty acids. *Biochim. Biophys. Acta* 62 509–518. 10.1016/0006-3002(62)90232-90239 13871487

[B21] Bouwstra J. A. Dubbelaar F. E. Gooris G. S. Ponec M. (2000). The lipid organisation in the skin barrier. *Acta Derm-venereol.* 80 23–30. 10.1080/000155500750042826 10884936

[B22] Bouwstra J. A. Gooris G. S. Spek J. A. van der Bras W. (1991). Structural investigations of human stratum corneum by small-angle x-ray scattering. *J. Invest. Dermatol.* 97 1005–1012. 10.1111/1523-1747.ep12492217 1748810

[B23] Bouwstra J. A. Gooris G. S. Vries M. A. S. van der Spek J. A. Bras W. (1992). Structure of human stratum corneum as a function of temperature and hydration: a wide-angle X-ray diffraction study. *Int. J. Pharmaceut.* 84 205–216. 10.1016/0378-5173(92)90158-x

[B24] Bouwstra J. Gooris G. Ponec M. (2002). The lipid organisation of the skin barrier: liquid and crystalline domains coexist in lamellar phases. *J. Biol. Phys.* 28 211–223. 10.1023/a:1019983715589 23345770 PMC3456653

[B25] Bowser P. A. Nugteren D. H. White R. J. Houtsmuller U. M. T. Prottey C. (1985). Identification, isolation and characterization of epidermal lipids containing linoleic acid. *Biochim. Biophys. Acta* 834 419–428. 10.1016/0005-2760(85)90016-90015 3995076

[B26] Breton J. Woolf D. Young P. Chabot-Fletcher M. (1996). Human keratinocytes lack the components to produce leukotriene B4. *J. Invest. Dermatol.* 106 162–167. 10.1111/1523-1747.ep12329890 8592068

[B27] Brogden K. A. Drake D. R. Dawson D. V. Hill J. R. Bratt C. L. Wertz P. W. (2011). “Antimicrobial lipids of the skin and oral mucosa,” in *Innate Immune System of Skin and Oral Mucosa: Properties and Impact in Pharmaceutics, Cosmetics, and Personal Care Products*, eds Dayan N. Wertz P. W. (Hoboken, NJ: Wiley).

[B28] Brogden N. K. Mehalick L. Fischer C. L. Wertz P. W. Brogden K. A. (2012). The emerging role of peptides and lipids as antimicrobial epidermal barriers and modulators of local inflammation. *Skin Pharmacol. Phys.* 25 167–181. 10.1159/000337927 22538862 PMC3564229

[B29] Bronfman M. Morales M. N. Orellana A. (1988). Diacylglycerol activation of protein kinase C is modulated by long-chain acyl-CoA. *Biochem. Biophys. Res. Co* 152 987–992. 10.1016/s0006-291x(88)80381-4 3377782

[B30] Burtenshaw J. M. L. (1942). The mechanism of self-disinfection of the human skin and its appendages. *J. Hyg-cambridge* 42 184–210. 10.1017/s0022172400035373 20475622 PMC2199810

[B31] Capolupo L. Khven I. Mazzeo L. Glousker G. Russo F. Montoya J. P. (2021). Sphingolipid control of fibroblast heterogeneity revealed by single-cell lipidomics. *Biorxiv [preprint]* 10.1101/2021.02.23.432420

[B32] Carson D. D. Daneo-Moore L. (1980). Effects of fatty acids on lysis of *Streptococcus faecalis*. *J. Bacteriol.* 141 1122–1126. 10.1128/jb.141.3.1122-1126.1980 6102557 PMC293793

[B33] Chamberlain N. R. Mehrtens B. G. Xiong Z. Kapral F. A. Boardman J. L. Rearick J. I. (1991). Correlation of carotenoid production, decreased membrane fluidity, and resistance to oleic acid killing in *Staphylococcus aureus* 18Z. *Infect. Immun.* 59 4332–4337. 10.1128/iai.59.12.4332-4337.1991 1937793 PMC259045

[B34] Champagne A. M. Muñoz-Garcia A. Shtayyeh T. Tieleman B. I. Hegemann A. Clement M. E. (2012). Lipid composition of the stratum corneum and cutaneous water loss in birds along an aridity gradient. *J. Exp. Biol.* 215 4299–4307. 10.1242/jeb.077016 22972881

[B35] Cho Y. Ziboh V. A. (1994a). Expression of protein kinase C isozymes in guinea pig epidermis: selective inhibition of PKC-beta activity by 13-hydroxyoctadecadienoic acid-containing diacylglycerol. *J. Lipid Res.* 35 913–921. 10.1016/s0022-2275(20)39185-39189 8071613

[B36] Cho Y. Ziboh V. A. (1994b). Incorporation of 13-hydroxyoctadecadienoic acid (13-HODE) into epidermal ceramides and phospholipids: phospholipase C-catalyzed release of novel 13-HODE-containing diacylglycerol. *J. Lipid Res.* 35 255–262. 10.1016/s0022-2275(20)41214-41213 8169529

[B37] Cho Y. Ziboh V. A. (1995). Nutritional modulation of guinea pig skin hyperproliferation by essential fatty acid deficiency is associated with selective down regulation of protein kinase C-beta. *J. Nutrition* 125 2741–2750. 7472653 10.1093/jn/125.11.2741

[B38] Cho Y. Ziboh V. A. (1997). A novel 15-hydroxyeicosatrienoic acid-substituted diacylglycerol (15-HETrE-DAG) selectively inhibits epidermal protein kinase C-β. *Biochim. Biophys. Acta* 1349 67–71. 10.1016/s0005-2760(97)00144-146 9421197

[B39] Choi H. Kim S. Kim H.-J. Kim K.-M. Lee C.-H. Shin J. H. (2010). Sphingosylphosphorylcholine down-regulates filaggrin gene transcription through NOX5-based NADPH oxidase and cyclooxygenase-2 in human keratinocytes. *Biochem. Pharmacol.* 80 95–103. 10.1016/j.bcp.2010.03.009 20230798

[B40] Connelly J. T. Gautrot J. E. Trappmann B. Tan D. W.-M. Donati G. Huck W. T. S. (2010). Actin and serum response factor transduce physical cues from the microenvironment to regulate epidermal stem cell fate decisions. *Nat. Cell Biol.* 12 711–718. 10.1038/ncb2074 20581838

[B41] Connelly J. T. Mishra A. Gautrot J. E. Watt F. M. (2011). Shape-Induced terminal differentiation of human epidermal stem cells requires p38 and is regulated by histone acetylation. *PLoS One* 6:e27259. 10.1371/journal.pone.0027259 22073300 PMC3206954

[B42] de Jager M. W. Gooris G. S. Dolbnya I. P. Bras W. Ponec M. Bouwstra J. A. (2003). The phase behaviour of skin lipid mixtures based on synthetic ceramides. *Chem. Phys. Lipids* 124 123–134. 10.1016/s0009-3084(03)00050-51 12818738

[B43] Demoyer J. S. Skalak T. C. Durieux M. E. (2000). Lysophosphatidic acid enhances healing of acute cutaneous wounds in the mouse. *Wound Repair Regen.* 8 530–537. 10.1046/j.1524-475x.2000.00530.x 11208180

[B44] Desbois A. P. Smith V. J. (2010). Antibacterial free fatty acids: activities, mechanisms of action and biotechnological potential. *Appl. Microbiol. Biot.* 85 1629–1642. 10.1007/s00253-009-2355-2353 19956944

[B45] Dietrich C. Bagatolli L. A. Volovyk Z. N. Thompson N. L. Levi M. Jacobson K. (2001). Lipid rafts reconstituted in model membranes. *Biophys. J.* 80 1417–1428. 10.1016/s0006-3495(01)76114-76110 11222302 PMC1301333

[B46] Doepping S. Funk C. D. Habenicht A. J. R. Spanbroek R. (2007). Selective 5-Lipoxygenase expression in langerhans cells and impaired dendritic cell migration in 5-LO-Deficient mice reveal leukotriene action in skin. *J. Invest. Dermatol.* 127 1692–1700. 10.1038/sj.jid.5700796 17392829

[B47] Dorschner R. A. Pestonjamasp V. K. Tamakuwala S. Ohtake T. Rudisill J. Nizet V. (2001). Cutaneous injury induces the release of cathelicidin anti-microbial peptides active against group a *Streptococcus*. *J. Invest. Dermatol.* 117 91–97. 10.1046/j.1523-1747.2001.01340.x 11442754

[B48] Dowd P. M. Black A. K. Woollard P. M. Camp R. D. R. Greaves M. W. (1985). Cutaneous responses to 12-Hydroxy-5,8,10,14-eicosatetraenoic Acid (12-HETE). *J. Invest. Dermatol.* 84 537–541. 10.1111/1523-1747.ep12273537 3998504

[B49] Downing D. T. Strauss J. S. (1974). Synthesis and composition of surface lipids of human skin. *J. Invest. Dermatol.* 62 228–244. 10.1111/1523-1747.ep12676793

[B50] Drake D. R. Brogden K. A. Dawson D. V. Wertz P. W. (2008). Thematic review series: skin lipids. antimicrobial lipids at the skin surface. *J. Lipid Res.* 49 4–11. 10.1194/jlr.r700016-jlr200 17906220

[B51] Dréno B. Dagnelie M. A. Khammari A. Corvec S. (2020). The skin microbiome: a new actor in inflammatory acne. *Am. J. Clin. Dermatol.* 21 18–24. 10.1007/s40257-020-00531-531 32910436 PMC7584556

[B52] Drochmans P. Freudenstein C. Wanson J.-C. Laurent L. Keenan T. W. Stadler J. (1978). Structure and biochemical composition of desmosomes and tonofilaments isolated from calf muzzle epidermis. *J. Cell Biol.* 79 427–443. 10.1083/jcb.79.2.427 569157 PMC2110254

[B53] Elias P. M. Friend D. S. (1975). The permeability barrier in mammalian epidermis. *J. Cell Biol.* 65 180–191. 10.1083/jcb.65.1.180 1127009 PMC2111161

[B54] Elias P. M. Goerke J. Friend D. S. (1977). Mammalian epidermal barrier layer lipids: composition and influence on structure. *J. Invest. Dermatol.* 69 535–546. 10.1111/1523-1747.ep12687968 925377

[B55] Elias P. M. Mauro T. Rassner U. Kömüves L. Brown B. E. Menon G. K. (1998). The secretory granular cell: the outermost granular cell as a specialized secretory cell. *J. Invest. Derm Symp. P* 3 87–100. 10.1038/jidsymp.1998.20 9734820

[B56] Elias P. M. Williams M. L. Holleran W. M. Jiang Y. J. Schmuth M. (2008). Thematic review series: skin lipids. pathogenesis of permeability barrier abnormalities in the ichthyoses: inherited disorders of lipid metabolism. *J. Lipid Res.* 49 697–714. 10.1194/jlr.r800002-jlr200 18245815 PMC2844331

[B57] Elias P. M. Williams M. L. Maloney M. E. Bonifas J. A. Brown B. E. Grayson S. (1984). Stratum corneum lipids in disorders of cornification. Steroid sulfatase and cholesterol sulfate in normal desquamation and the pathogenesis of recessive X-linked ichthyosis. *J. Clin. Invest.* 74 1414–1421. 10.1172/jci111552 6592175 PMC425309

[B58] Falconer A. Ikram M. Bissett C. E. Cerio R. Quinn A. G. Ali R. S. (2001). Expression of the peptide antibiotics human β Defensin-1 and Human β Defensin-2 in normal human skin. *J. Invest. Dermatol.* 117 106–111. 10.1046/j.0022-202x.2001.01401.x 11442756

[B59] Fischer C. L. (2020). Antimicrobial activity of host-derived lipids. *Antibiotics* 9:75. 10.3390/antibiotics9020075 32054068 PMC7168235

[B60] Fischer C. L. Drake D. R. Dawson D. V. Blanchette D. R. Brogden K. A. Wertz P. W. (2012a). Antibacterial activity of sphingoid bases and fatty acids against gram-positive and gram-negative bacteria. *Antimicrob Agents Ch* 56 1157–1161. 10.1128/aac.05151-5111 PMC3294957 22155833

[B61] Fischer C. L. Walters K. S. Drake D. R. Blanchette D. R. Dawson D. V. Brogden K. A. (2012b). Sphingoid bases are taken up by *Escherichia coli* and *Staphylococcus aureus* and induce ultrastructural damage. *Skin Pharmacol. Phys.* 26 36–44. 10.1159/000343175 23128426 PMC3634627

[B62] Fluhr J. W. Kao J. Ahn S. K. Feingold K. R. Elias P. M. Jain M. (2001). Generation of free fatty acids from phospholipids regulates stratum corneum acidification and integrity. *J. Invest. Dermatol.* 117 44–51. 10.1046/j.0022-202x.2001.01399.x 11442748

[B63] Forslind B. Lindberg M. Malmqvist K. G. Pallon J. Roomans G. M. Werner-Linde Y. (1995). Human skin physiology studied by particle probe microanalysis. *Scann. Microscopy* 9 1011–1025. 8819884

[B64] Freinkel R. K. Traczyk T. N. (1985). Lipid composition and acid hydrolase content of lamellar granules of fetal rat epidermis. *J. Invest. Dermatol.* 85 295–298. 10.1111/1523-1747.ep12276831 4045218

[B65] Fulton C. Anderson G. M. Zasloff M. Bull R. Quinn A. G. (1997). Expression of natural peptide antibiotics in human skin. *Lancet* 350 1750–1751. 9413472 10.1016/S0140-6736(05)63574-X

[B66] Galbraith H. Miller T. B. (1973). Effect of long chain fatty acids on bacterial respiration and amino acid uptake. *J. Appl. Bacteriol.* 36 659–675. 10.1111/j.1365-2672.1973.tb04151.x 4787613

[B67] Gallo R. L. Nakatsuji T. (2011). Microbial symbiosis with the innate immune defense system of the skin. *J. Invest. Dermatol.* 131 1974–1980. 10.1038/jid.2011.182 21697881 PMC3174284

[B68] Geraldo L. H. M. de Spohr T. C. L. S. do Amaral R. F. da Fonseca A. C. C. Garcia C. de Mendes F. A. (2021). Role of lysophosphatidic acid and its receptors in health and disease: novel therapeutic strategies. *Signal Trans. Target Ther.* 6:45. 10.1038/s41392-020-00367-365 PMC7851145 33526777

[B69] Gibson D. F. C. Ratnam A. V. Bikle D. D. (1996). Evidence for separate control mechanisms at the message, protein, and enzyme activation levels for transglutaminase during calcium-induced differentiation of normal and transformed human keratinocytes. *J. Invest. Dermatol.* 106 154–161. 10.1111/1523-1747.ep12329856 8592067

[B70] Gomez-Larrauri A. Presa N. Dominguez-Herrera A. Ouro A. Trueba M. Gomez-Muñoz A. (2020). Role of bioactive sphingolipids in physiology and pathology. *Essays Biochem.* 64 579–589. 10.1042/ebc20190091 32579188

[B71] Götz F. Verheij H. M. Rosenstein R. (1998). Staphylococcal lipases: molecular characterisation, secretion, and processing. *Chem. Phys. Lipids* 93 15–25. 10.1016/s0009-3084(98)00025-25 9720246

[B72] Gray G. M. King I. A. Yardley H. J. (1980). The plasma membrane of Malpighian cells from pig epidermis: isolation and lipid and protein composition. *Br. J. Dermatol.* 103 505–516. 10.1111/j.1365-2133.1980.tb01665.x 7437317

[B73] Grayson S. Johnson-Winegar A. G. Wintroub B. U. Isseroff R. R. Epstein E. H. Elias P. M. (1985). Lamellar body-enriched fractions from neonatal mice: preparative techniques and partial characterization. *J. Invest. Dermatol.* 85 289–294. 10.1111/1523-1747.ep12276826 4045217

[B74] Greenway D. L. A. Dyke K. G. H. (1979). Mechanism of the inhibitory action of linoleic acid on the growth of *Staphylococcus aureus*. *Microbiology* 115 233–245. 10.1099/00221287-115-1-233 93615

[B75] Grice E. A. Segre J. A. (2011). The skin microbiome. *Nat. Rev. Microbiol.* 9 244–253. 10.1038/nrmicro2537 21407241 PMC3535073

[B76] Grice E. A. Kong H. H. Conlan S. Deming C. B. Davis J. Young A. C. (2009). Topographical and temporal diversity of the human skin microbiome. *Science* 324 1190–1192. 10.1126/science.1171700 19478181 PMC2805064

[B77] Grond S. Eichmann T. O. Dubrac S. Kolb D. Schmuth M. Fischer J. (2017). PNPLA1 deficiency in mice and humans leads to a defect in the synthesis of Omega-O-Acylceramides. *J. Invest. Dermatol.* 137 394–402. 10.1016/j.jid.2016.08.036 27751867 PMC5298181

[B78] Gutknecht J. (1988). Proton conductance caused by long-chain fatty acids in phospholipid bilayer membranes. *J. Membr. Biol.* 106 83–93. 10.1007/bf01871769 2852256

[B79] Gutteridge J. M. C. Lamport P. Dormandy T. L. (1974). Autoxidation as a cause of antibacterial activity in unsaturated fatty acids. *J. Med. Microbiol.* 7 387–389. 10.1099/00222615-7-3-387 4371778

[B80] Hachem J.-P. Crumrine D. Fluhr J. Brown B. E. Feingold K. R. Elias P. M. (2003). pH directly regulates epidermal permeability barrier homeostasis, and stratum corneum integrity/cohesion. *J. Invest. Dermatol.* 121 345–353. 10.1046/j.1523-1747.2003.12365.x 12880427

[B81] Hanley K. Jiang Y. He S. S. Friedman M. Elias P. M. Bikle D. D. (1998). Keratinocyte differentiation is stimulated by activators of the nuclear hormone receptor PPARα. *J. Invest. Dermatol.* 110 368–375. 10.1046/j.1523-1747.1998.00139.x 9540977

[B82] Hanley K. Kömüves L. G. Ng D. C. Schoonjans K. He S. S. Lau P. (2000a). Farnesol stimulates differentiation in epidermal keratinocytes *via* PPARα*. *J. Biol. Chem.* 275 11484–11491. 10.1074/jbc.275.15.11484 10753967

[B83] Hanley K. Ng D. C. He S. Lau P. Min K. Elias P. M. (2000b). Oxysterols induce differentiation in human keratinocytes and increase Ap-1-Dependent involucrin transcription. *J. Invest. Dermatol.* 114 545–553. 10.1046/j.1523-1747.2000.00895.x 10692116

[B84] Hannun Y. A. (1994). The sphingomyelin cycle and the second messenger function of ceramide. *J. Biol. Chem.* 269 3125–3128. 10.1016/s0021-9258(17)41834-41835 8106344

[B85] Hannun Y. A. Obeid L. M. (2008). Principles of bioactive lipid signalling: lessons from sphingolipids. *Nat. Rev. Mol. Cell Biol.* 9 139–150. 10.1038/nrm2329 18216770

[B86] Hannun Y. A. Loomis C. R. Merrill A. H. Jr. Bell R. M. (1986). Sphingosine inhibition of protein kinase C activity and of phorbol dibutyrate binding in vitro and in human platelets. *J. Biol. Chem.* 261 12604–12609. 3462188

[B87] Hannun Y. A. Luberto C. Argraves K. M. (2001). Enzymes of sphingolipid metabolism: from modular to integrative signaling ^†^. *Biochemistry* 40 4893–4903. 10.1021/bi002836k 11305904

[B88] Hanyu O. Nakae H. Miida T. Higashi Y. Fuda H. Endo M. (2012). Cholesterol sulfate induces expression of the skin barrier protein filaggrin in normal human epidermal keratinocytes through induction of RORα. *Biochem. Biophy. Res. Commun.* 428 99–104. 10.1016/j.bbrc.2012.10.013 23063684

[B89] Hattori T. Obinata H. Ogawa A. Kishi M. Tatei K. Ishikawa O. (2008). G2A plays proinflammatory roles in human keratinocytes under oxidative stress as a receptor for 9-hydroxyoctadecadienoic acid. *J. Invest. Dermatol.* 128 1123–1133. 10.1038/sj.jid.5701172 18034171

[B90] Higuchi K. Kawashima M. Takagi Y. Kondo H. Yada Y. Ichikawa Y. (2001). Sphingosylphosphorylcholine is an activator of transglutaminase activity in human keratinocytes. *J. Lipid Res.* 42 1562–1570. 10.1016/s0022-2275(20)32209-32204 11590211

[B91] Hirai H. Tanaka K. Yoshie O. Ogawa K. Kenmotsu K. Takamori Y. (2001). Prostaglandin D2 selectively induces chemotaxis in T Helper Type 2 Cells, eosinophils, and basophils *via* seven-transmembrane receptor Crth2. *J. Exp. Med.* 193 255–262. 10.1084/jem.193.2.255 11208866 PMC2193345

[B92] Hiratsuka T. Bordeu I. Pruessner G. Watt F. M. (2020). Regulation of ERK basal and pulsatile activity control proliferation and exit from the stem cell compartment in mammalian epidermis. *Proc. Natl. Acad. Sci. U S A.* 117 17796–17807. 10.1073/pnas.2006965117 32651268 PMC7395546

[B93] Hong J. H. Youm J.-K. Kwon M. J. Park B. D. Lee Y.-M. Lee S.-I. (2008). K6PC-5, a direct activator of sphingosine kinase 1, promotes epidermal differentiation through intracellular Ca2+ signaling. *J. Invest. Dermatol.* 128 2166–2178. 10.1038/jid.2008.66 18385762

[B94] Huang W.-C. Tsai T.-H. Chuang L.-T. Li Y.-Y. Zouboulis C. C. Tsai P.-J. (2014). Anti-bacterial and anti-inflammatory properties of capric acid against Propionibacterium acnes: a comparative study with lauric acid. *J. Dermatol. Sci.* 73 232–240. 10.1016/j.jdermsci.2013.10.010 24284257

[B95] Igawa S. Choi J. E. Wang Z. Chang Y.-L. Wu C.-C. Werbel T. (2019). Human keratinocytes use sphingosine 1-Phosphate and its receptors to communicate *Staphylococcus aureus* invasion and activate host defense. *J. Invest. Dermatol.* 139 1743–1752.e5. 10.1016/j.jid.2019.02.010. 30807768 PMC7682680

[B96] Ikenouchi J. (2018). Roles of membrane lipids in the organization of epithelial cells: old and new problems. *Tissue Barriers* 6 1–8. 10.1080/21688370.2018.1502531 30156967 PMC6179127

[B97] Imokawa G. Takagi Y. Higuchi K. Kondo H. Yada Y. (1999). Sphingosylphosphorylcholine is a potent inducer of intercellular adhesion Molecule-1 expression in human keratinocytes. *J. Invest. Dermatol.* 112 91–96. 10.1046/j.1523-1747.1999.00462.x 9886270

[B98] Irving L. Schmidt-Nielsen K. Abrahamsen N. S. B. (1957). On the melting points of animal fats in cold climates. *Physiol. Zool.* 30 93–105. 10.1086/physzool.30.2.30155356

[B99] Janes S. M. Ofstad T. A. Campbell D. H. Eddaoudi A. Warnes G. Davies D. (2009). PI3-kinase-dependent activation of apoptotic machinery occurs on commitment of epidermal keratinocytes to terminal differentiation. *Cell Res.* 19 328–339. 10.1038/cr.2008.281 18766172 PMC2650684

[B100] Janes S. M. Ofstad T. A. Campbell D. H. Watt F. M. Prowse D. M. (2004). Transient activation of FOXN1 in keratinocytes induces a transcriptional programme that promotes terminal differentiation: contrasting roles of FOXN1 and Akt. *J. Cell Sci.* 117 4157–4168. 10.1242/jcs.01302 15316080

[B101] Janowski B. A. Willy P. J. Devi T. R. Falck J. R. Mangelsdorf D. J. (1996). An oxysterol signalling pathway mediated by the nuclear receptor LXRα. *Nature* 383 728–731. 10.1038/383728a0 8878485

[B102] Janssen-Timmen U. Vickers P. J. Wittig U. Lehmann W. D. Stark H.-J. Fusenig N. E. (1995). Expression of 5-lipoxygenase in differentiating human skin keratinocytes. *Proc. Natl. Acad. Sci. U S A.* 92 6966–6970. 10.1073/pnas.92.15.6966 7624354 PMC41452

[B103] Jeon S. Song J. Lee D. Kim G.-T. Park S.-H. Shin D.-Y. (2020). Inhibition of sphingosine 1-phosphate lyase activates human keratinocyte differentiation and attenuates psoriasis in mice. *J. Lipid Res.* 61 20–32. 10.1194/jlr.ra119000254 31690639 PMC6939600

[B104] Jiang Y. J. Kim P. Uchida Y. Elias P. M. Bikle D. D. Grunfeld C. (2013). Ceramides stimulate caspase-14 expression in human keratinocytes. *Exp. Dermatol.* 22 113–118. 10.1111/exd.12079 23362869 PMC4099380

[B105] Jiang Y. J. Lu B. Kim P. Paragh G. Schmitz G. Elias P. M. (2008). PPAR and LXR activators regulate ABCA12 expression in human keratinocytes. *J. Invest. Dermatol.* 128 104–109. 10.1038/sj.jid.5700944 17611579

[B106] Jiang Y. J. Lu B. Tarling E. J. Kim P. Man M.-Q. Crumrine D. (2010). Regulation of ABCG1 expression in human keratinocytes and murine epidermis[S]. *J. Lipid Res.* 51 3185–3195. 10.1194/jlr.m006445 20675829 PMC2952559

[B107] Jiang Y. J. Uchida Y. Lu B. Kim P. Mao C. Akiyama M. (2009). Ceramide stimulates ABCA12 expression *via* peroxisome proliferator-activated receptor δ in human keratinocytes*. *J. Biol. Chem.* 284 18942–18952. 10.1074/jbc.m109.006973 19429679 PMC2707228

[B108] Kabara J. J. Swieczkowski D. M. Conley A. J. Truant J. P. (1972). Fatty acids and derivatives as antimicrobial agents. *Antimicrob Agents Chemother* 2 23–28. 10.1128/aac.2.1.23 4670656 PMC444260

[B109] Kabara J. J. Vrable R. Jie M. S. F. L. K. (1977). Antimicrobial lipids: natural and synthetic fatty acids and monoglycerides. *Lipids* 12 753–759. 10.1007/bf02570908 409896

[B110] Kabashima K. Nagamachi M. Honda T. Nishigori C. Miyachi Y. Tokura Y. (2007). Prostaglandin E2 is required for ultraviolet B-induced skin inflammation *via* EP2 and EP4 receptors. *Lab. Invest.* 87 49–55. 10.1038/labinvest.3700491 17075575

[B111] Kabashima K. Sakata D. Nagamachi M. Miyachi Y. Inaba K. Narumiya S. (2003). Prostaglandin E2-EP4 signaling initiates skin immune responses by promoting migration and maturation of Langerhans cells. *Nat. Med.* 9 744–749. 10.1038/nm872 12740571

[B112] Kalinin A. E. Kajava A. V. Steinert P. M. (2002). Epithelial barrier function: assembly and structural features of the cornified cell envelope. *Bioessays* 24 789–800. 10.1002/bies.10144 12210515

[B113] Kanda N. Ishikawa T. Watanabe S. (2010). Prostaglandin D2 induces the production of human β-defensin-3 in human keratinocytes. *Biochem. Pharmacol.* 79 982–989. 10.1016/j.bcp.2009.11.012 19925780

[B114] Karsak M. Gaffal E. Date R. Wang-Eckhardt L. Rehnelt J. Petrosino S. (2007). Attenuation of allergic contact dermatitis through the endocannabinoid system. *Science* 316 1494–1497. 10.1126/science.1142265 17556587

[B115] Kendall A. C. Nicolaou A. (2013). Bioactive lipid mediators in skin inflammation and immunity. *Prog. Lipid Res.* 52 141–164. 10.1016/j.plipres.2012.10.003 23124022

[B116] Khnykin D. Miner J. H. Jahnsen F. (2011). Role of fatty acid transporters in epidermis. *Dermato-endocrinology* 3 53–61. 10.4161/derm.3.2.14816 21695012 PMC3117002

[B117] Kim D. Kim S. Moon S. Chung J. Kim K. Cho K. (2001). Ceramide inhibits cell proliferation through Akt/PKB inactivation and decreases melanin synthesis in Mel-Ab Cells. *Pigm Cell Res.* 14 110–115. 10.1034/j.1600-0749.2001.140206.x 11310790

[B118] Kim D.-S. Kim S.-Y. Kleuser B. Schäfer-Korting M. Kim K.-H. Park K.-C. (2004). Sphingosine-1-phosphate inhibits human keratinocyte proliferation *via* Akt/protein kinase B inactivation. *Cell. Signal.* 16 89–95. 10.1016/s0898-6568(03)00114-111 14607279

[B119] Kim S.-N. Akindehin S. Kwon H.-J. Son Y.-H. Saha A. Jung Y.-S. (2018). Anti-inflammatory role of 15-lipoxygenase contributes to the maintenance of skin integrity in mice. *Sci. Rep.* 8:8856. 10.1038/s41598-018-27221-27227 PMC5995961 29891910

[B120] Kim Y.-I. Park K. Kim J. Y. Seo H. S. Shin K.-O. Lee Y.-M. (2014). An endoplasmic reticulum stress-initiated sphingolipid metabolite, Ceramide-1-Phosphate, regulates epithelial innate immunity by stimulating β-Defensin production. *Mol. Cell. Biol.* 34 4368–4378. 10.1128/mcb.00599-514 25312644 PMC4248733

[B121] Klee S. K. Farwick M. Lersch P. (2007). “Acne and its therapy,” in *Basic and Clinical Dermatology*, ed. Webster G. F. Rawlings A. V. 155–165. 10.3109/9781420018417.013

[B122] Knapp H. R. Melly M. A. (1986). Bactericidal effects of polyunsaturated fatty acids. *J. Infect. Dis.* 154 84–94. 10.1093/infdis/154.1.84 3086465

[B123] Kobayashi T. Voisin B. Kim D. Y. Kennedy E. A. Jo J.-H. Shih H.-Y. (2019). Homeostatic control of sebaceous glands by innate lymphoid cells regulates commensal bacteria equilibrium. *Cell* 176 982–997.e16. 10.1016/j.cell.2018.12.031. 30712873 PMC6532063

[B124] Köberlin M. S. Snijder B. Heinz L. X. Baumann C. L. Fauster A. Vladimer G. I. (2015). A conserved circular network of coregulated lipids modulates innate immune responses. *Cell* 162 170–183. 10.1016/j.cell.2015.05.051 26095250 PMC4523684

[B125] Kodicek E. Worden A. N. (1945). The effect of unsaturated fatty acids on *Lactobacillus helveticus* and other Gram-positive micro-organisms. *Biochem. J.* 39 78–85. 10.1042/bj0390078 16747862 PMC1258155

[B126] Konger R. L. Billings S. D. Thompson A. B. Morimiya A. Ladenson J. H. Landt Y. (2005a). Immunolocalization of low-affinity prostaglandin E2 receptors, EP1 and EP2, in adult human epidermis. *J. Invest. Dermatol.* 124 965–970. 10.1111/j.0022-202x.2005.23658.x 15854037

[B127] Konger R. L. Brouxhon S. Partillo S. VanBuskirk J. Pentland A. P. (2005b). The EP3 receptor stimulates ceramide and diacylglycerol release and inhibits growth of primary keratinocytes. *Exp. Dermatol.* 14 914–922. 10.1111/j.1600-0625.2005.00381.x 16274459

[B128] Konger R. L. Malaviya R. Pentland A. P. (1998). Growth regulation of primary human keratinocytes by prostaglandin E receptor EP2 and EP3 subtypes. *Biochim. Biophys. Acta* 1401 221–234. 9531979 10.1016/s0167-4889(97)00114-6

[B129] Kooyman D. J. (1932). Lipids of the skin. Some changes in the lipids of the epidermis during the process of keratinization. *Arch. Dermatol. Syph.* 25 444–450. 10.1001/archderm.1932.01450020460003

[B130] Ladd P. A. Du L. Capdevila J. H. Mernaugh R. Keeney D. S. (2003). Epoxyeicosatrienoic acids activate transglutaminases in situ and induce cornification of epidermal keratinocytes*. *J. Biol. Chem.* 278 35184–35192. 10.1074/jbc.m301666200 12840027

[B131] Lampe M. A. Williams M. L. Elias P. M. (1983). Human epidermal lipids: characterization and modulations during differentiation. *J. Lipid Res.* 24 131–140. 6833890

[B132] Landau J. W. (1983). Commentary: undecylenic acid and fungous infections. *Arch. Dermatol.* 119 351–353. 10.1001/archderm.1983.01650280079021 6340617

[B133] Landmann L. (1986). Epidermal permeability barrier: transformation of lamellar granule-disks into intercellular sheets by a membrane-fusion process, a freeze-fracture study. *J. Invest. Dermatol.* 87 202–209. 10.1111/1523-1747.ep12695343 3734471

[B134] Lee D.-Y. Huang C.-M. Nakatsuji T. Thiboutot D. Kang S.-A. Monestier M. (2009). Histone H4 is a major component of the antimicrobial action of human sebocytes. *J. Invest. Dermatol.* 129 2489–2496. 10.1038/jid.2009.106 19536143 PMC2765036

[B135] Lei L. Su J. Chen J. Chen W. Chen X. Peng C. (2018). The role of lysophosphatidic acid in the physiology and pathology of the skin. *Life Sci.* 220 194–200. 10.1016/j.lfs.2018.12.040 30584899

[B136] Leonti M. Casu L. Raduner S. Cottiglia F. Floris C. Altmann K.-H. (2010). Falcarinol is a covalent cannabinoid CB1 receptor antagonist and induces pro-allergic effects in skin. *Biochem. Pharmacol.* 79 1815–1826. 10.1016/j.bcp.2010.02.015 20206138

[B137] Levental I. Levental K. R. Heberle F. A. (2020). Lipid rafts: controversies resolved, mysteries remain. *Trends Cell Biol.* 30 341–353. 10.1016/j.tcb.2020.01.009 32302547 PMC7798360

[B138] Lewis R. A. Soter N. A. Diamond P. T. Austen K. F. Oates J. A. Roberts L. J. (1982). Prostaglandin D2 generation after activation of rat and human mast cells with anti-IgE. *J. Immunol.* 129 1627–1631. 6809826

[B139] Li Z. Cheng S. Lin Q. Cao W. Yang J. Zhang M. (2021). Single-cell lipidomics with high structural specificity by mass spectrometry. *Nat. Commun.* 12:2869. 10.1038/s41467-021-23161-23165 PMC8129106 34001877

[B140] Liakath-Ali K. Vancollie V. E. Lelliott C. J. Speak A. O. Lafont D. Protheroe H. J. (2016). Alkaline ceramidase 1 is essential for mammalian skin homeostasis and regulating whole-body energy expenditure. *J. Pathol.* 239 374–383. 10.1002/path.4737 27126290 PMC4924601

[B141] Lichte K. Rossi R. Danneberg K. ter Braak M. Kürschner U. Jakobs K. H. (2008). Lysophospholipid receptor-mediated calcium signaling in human keratinocytes. *J. Invest. Dermatol.* 128 1487–1498. 10.1038/sj.jid.5701207 18172456

[B142] Long V. J. W. (1970). Variations in lipid composition at different depths in the cow snout epidermis. *J. Invest. Dermatol.* 55 269–273. 10.1111/1523-1747.ep12259974 5471890

[B143] Lovászi M. Szegedi A. Zouboulis C. C. Törõcsik D. (2017). Sebaceous-immunobiology is orchestrated by sebum lipids. *Dermato-endocrinology* 9:e1375636. 10.1080/19381980.2017.1375636 29484100 PMC5821166

[B144] Lu B. Jiang Y. J. Kim P. Moser A. Elias P. M. Grunfeld C. (2010). Expression and regulation of GPAT isoforms in cultured human keratinocytes and rodent epidermis. *J. Lipid Res.* 51 3207–3216. 10.1194/jlr.m007054 20719759 PMC2952561

[B145] Maccarrone M. Rienzo M. D. Battista N. Gasperi V. Guerrieri P. Rossi A. (2003). The endocannabinoid system in human keratinocytes - evidence that anandamide inhibits epidermal differentiation through CB1 receptor-dependent inhibition of Protein Kinase C, Activating Protein-1, and transglutaminase. *J. Biol. Chem.* 278 33896–33903. 10.1074/jbc.m303994200 12815050

[B146] Maciejewski-Lenoir D. Richman J. G. Hakak Y. Gaidarov I. Behan D. P. Connolly D. T. (2006). Langerhans cells release prostaglandin D2 in response to nicotinic acid. *J. Invest. Dermatol.* 126 2637–2646. 10.1038/sj.jid.5700586 17008871

[B147] Madison K. C. Swartzendruber D. C. Wertz P. W. Downing D. T. (1987). Presence of intact intercellular lipid lamellae in the upper layers of the stratum corneum. *J. Invest. Dermatol.* 88 714–718. 10.1111/1523-1747.ep12470386 3585055

[B148] Man M.-Q. Choi E.-H. Schmuth M. Crumrine D. Uchida Y. Elias P. M. (2006). Basis for improved permeability barrier homeostasis induced by PPAR and LXR activators: liposensors stimulate lipid synthesis, lamellar body secretion, and post-secretory lipid processing. *J. Invest. Dermatol.* 126 386–392. 10.1038/sj.jid.5700046 16374473

[B149] Mao-Qiang M. Fowler A. J. Schmuth M. Lau P. Chang S. Brown B. E. (2004). Peroxisome-Proliferator-Activated Receptor (PPAR)-γ activation stimulates keratinocyte differentiation. *J. Invest. Dermatol.* 123 305–312. 10.1111/j.0022-202x.2004.23235.x 15245430

[B150] Marples R. R. Downing D. T. Kligman A. M. (1971). Control of free fatty acids in human surface lipids by corynebacterium acnes. *J. Invest. Dermatol.* 56 127–131. 10.1111/1523-1747.ep12260695 4997367

[B151] Marsh N. L. Elias P. M. Holleran W. M. (1995). Glucosylceramides stimulate murine epidermal hyperproliferation. *J. Clin. Invest.* 95 2903–2909. 10.1172/jci117997 7769132 PMC295978

[B152] Masukawa Y. Narita H. Sato H. Naoe A. Kondo N. Sugai Y. (2009). Comprehensive quantification of ceramide species in human stratum corneum. *J. Lipid Res.* 50 1708–1719. 10.1194/jlr.d800055-jlr200 19349641 PMC2724059

[B153] Matoltsy A. G. Parakkal P. F. (1965). Membrane-Coating granules of keratinizing epithelia. *J. Cell Biol.* 24 297–307. 10.1083/jcb.24.2.297 14326115 PMC2106574

[B154] Mazereeuw-Hautier J. Gres S. Fanguin M. Cariven C. Fauvel J. Perret B. (2005). Production of lysophosphatidic acid in blister fluid: involvement of a lysophospholipase D activity. *J. Invest. Dermatol.* 125 421–427. 10.1111/j.0022-202x.2005.23855.x 16117781 PMC1885457

[B155] Mishra A. Oules B. Pisco A. O. Ly T. Liakath-Ali K. Walko G. (2017). A protein phosphatase network controls the temporal and spatial dynamics of differentiation commitment in human epidermis. *eLife* 6:e27356. 10.7554/elife.27356 29043977 PMC5667932

[B156] Mobasseri S. A. Zijl S. Salameti V. Walko G. Stannard A. Garcia-Manyes S. (2019). Patterning of human epidermal stem cells on undulating elastomer substrates reflects differences in cell stiffness. *Acta Biomater.* 87 256–264. 10.1016/j.actbio.2019.01.063 30710711 PMC6401207

[B157] Molleyres L. P. Rando R. R. (1988). Structural studies on the diglyceride-mediated activation of protein kinase C. *J. Biol. Chem.* 263 14832–14838. 3170567

[B158] Mori T. Takai Y. Yu B. Takahashi J. Nishizuka Y. Fujikura T. (1982). Specificity of the fatty acyl moieties of diacylglycerol for the activation of calcium-activated, phospholipid-dependent protein Kinase1. *J. Biochem.* 91 427–432. 6461644 10.1093/oxfordjournals.jbchem.a133714

[B159] Müller H. Hofer S. Kaneider N. Neuwirt H. Mosheimer B. Mayer G. (2005). The immunomodulator FTY720 interferes with effector functions of human monocyte-derived dendritic cells. *Eur. J. Immunol.* 35 533–545. 10.1002/eji.200425556 15657952

[B160] Muro E. Atilla-Gokcumen G. E. Eggert U. S. (2014). Lipids in cell biology: how can we understand them better? *Mol. Biol. Cell* 25 1819–1823. 10.1091/mbc.e13-09-0516 24925915 PMC4055261

[B161] Nakatsuji T. Kao M. C. Fang J.-Y. Zouboulis C. C. Zhang L. Gallo R. L. (2009). Antimicrobial property of lauric acid against propionibacterium acnes: its therapeutic potential for inflammatory acne vulgaris. *J. Invest. Dermatol.* 129 2480–2488. 10.1038/jid.2009.93 19387482 PMC2772209

[B162] Natsuga K. Cipolat S. Watt F. M. (2016). Increased bacterial load and expression of antimicrobial peptides in skin of barrier-deficient mice with reduced cancer susceptibility. *J. Invest. Dermatol.* 136 99–106. 10.1038/jid.2015.383 26763429 PMC4759621

[B163] Nekrasova O. Harmon R. M. Broussard J. A. Koetsier J. L. Godsel L. M. Fitz G. N. (2018). Desmosomal cadherin association with Tctex-1 and cortactin-Arp2/3 drives perijunctional actin polymerization to promote keratinocyte delamination. *Nat. Commun.* 9:1053. 10.1038/s41467-018-03414-3416 PMC5849617 29535305

[B164] Nemes Z. Steinert P. M. (1999). Bricks and mortar of the epidermal barrier. *Exp. Mol. Med.* 31 5–19. 10.1038/emm.1999.2 10231017

[B165] Nemes Z. Marekov L. N. Fésüs L. Steinert P. M. (1999). A novel function for transglutaminase 1: attachment of long-chain ω-hydroxyceramides to involucrin by ester bond formation. *Proc. Natl. Acad. Sci. U S A.* 96 8402–8407. 10.1073/pnas.96.15.8402 10411887 PMC17528

[B166] Nicolaides N. (1974). Skin lipids: their biochemical uniqueness. *Science* 186 19–26. 10.1126/science.186.4158.19 4607408

[B167] Nicolaides N. Wells G. C. (1957). On the biogenesis of the free fatty acids in human skin surface fat*. *J. Invest. Dermatol.* 29 423–433. 10.1038/jid.1957.118 13502598

[B168] Nicolaides N. Fu H. C. Rice G. R. (1968). The skin surface lipids of man compared with those of eighteen species of animals. *J. Invest. Dermatol.* 51 83–89. 10.1038/jid.1968.96 4980329

[B169] Nikaido H. (1976). Outer membrane of *Salmonella typhimurium* transmembrane diffusion of some hydrophobic substances. *Biochim. Biophys. Acta* 433 118–132. 769835 10.1016/0005-2736(76)90182-6

[B170] Nikkari T. (1974). Comparative chemistry of sebum. *J. Invest. Dermatol.* 62 257–267. 10.1111/1523-1747.ep12676800 4206501

[B171] Nishizuka Y. (1992). Intracellular signaling by hydrolysis of phospholipids and activation of protein kinase C. *Science* 258 607–614. 10.1126/science.1411571 1411571

[B172] Nixon G. F. Mathieson F. A. Hunter I. (2008). The multi-functional role of sphingosylphosphorylcholine. *Prog. Lipid Res.* 47 62–75. 10.1016/j.plipres.2007.11.001 18042469

[B173] Norlén L. Al-Amoudi A. Dubochet J. (2003). A cryotransmission electron microscopy study of skin barrier formation. *J. Invest. Dermatol.* 120 555–560. 10.1046/j.1523-1747.2003.12102.x 12648217

[B174] Odland G. F. (1960). A submicroscopic granular component in human epidermis* * from the department of anatomy, University of Washington, Seattle, Washington. *J. Invest. Dermatol.* 34 11–15. 10.1038/jid.1960.4 14428292

[B175] Ogawa E. Owada Y. Ikawa S. Adachi Y. Egawa T. Nemoto K. (2011). Epidermal FABP (FABP5) regulates keratinocyte differentiation by 13(S)-HODE-mediated activation of the NF-κB signaling pathway. *J. Invest. Dermatol.* 131 604–612. 10.1038/jid.2010.342 21068754

[B176] Oizumi A. Nakayama H. Okino N. Iwahara C. Kina K. Matsumoto R. (2014). *Pseudomonas*-Derived ceramidase induces production of inflammatory mediators from human keratinocytes *via* Sphingosine-1-Phosphate. *PLoS One* 9:e89402. 10.1371/journal.pone.0089402 24586752 PMC3934885

[B177] Opálka L. Kovaàčik A. Maixner J. Vaìvrovaì K. (2016). Omega-O-Acylceramides in skin lipid membranes: effects of concentration, sphingoid base, and model complexity on microstructure and permeability. *Langmuir* 32 12894–12904. 10.1021/acs.langmuir.6b03082 27934529

[B178] Oulès B. Philippeos C. Segal J. Tihy M. Rudan M. V. Cujba A.-M. (2020). Contribution of GATA6 to homeostasis of the human upper pilosebaceous unit and acne pathogenesis. *Nat. Commun.* 11:5067. 10.1038/s41467-020-18784-z 33082341 PMC7575575

[B179] Paller A. S. Arnsmeier S. L. Alvarez-Franco M. Bremer E. G. (1993). Ganglioside GM3 inhibits the proliferation of cultured keratinocytes. *J. Invest. Dermatol.* 100 841–845. 10.1111/1523-1747.ep12476755 8496625

[B180] Pappas A. (2009). Epidermal surface lipids. *Dermato-endocrinology* 1 72–76. 10.4161/derm.1.2.7811 20224687 PMC2835894

[B181] Park K. Elias P. M. Shin K.-O. Lee Y.-M. Hupe M. Borkowski A. W. (2013). A novel role of a lipid species, Sphingosine-1-Phosphate, in epithelial innate immunity. *Mol. Cell. Biol.* 33 752–762. 10.1128/mcb.01103-1112 23230267 PMC3571353

[B182] Park K. Ikushiro H. Seo H. S. Shin K.-O. Kim Y. Kim J. Y. (2016). ER stress stimulates production of the key antimicrobial peptide, cathelicidin, by forming a previously unidentified intracellular S1P signaling complex. *Proc. Natl. Acad. Sci. U S A.* 113 E1334–E1342. 10.1073/pnas.1504555113 26903652 PMC4791017

[B183] Pavicic T. Wollenweber U. Farwick M. Korting H. C. (2007). Anti-microbial and -inflammatory activity and efficacy of phytosphingosine: an in vitro and in vivo study addressing acne vulgaris. *Int. J. Cosmetic Sci.* 29 181–190. 10.1111/j.1467-2494.2007.00378.x 18489348

[B184] Peters F. Tellkamp F. Brodesser S. Wachsmuth E. Tosetti B. Karow U. (2020). Murine epidermal ceramide synthase 4 is a key regulator of skin barrier homeostasis. *J. Invest. Dermatol.* 140 1927–1937.e5. 10.1016/j.jid.2020.02.006. 32092351

[B185] Peters F. Vorhagen S. Brodesser S. Jakobshagen K. Brüning J. C. Niessen C. M. (2015). Ceramide synthase 4 regulates stem cell homeostasis and hair follicle cycling. *J. Invest. Dermatol.* 135 1501–1509. 10.1038/jid.2015.60 25705848

[B186] Piazza G. A. Ritter J. L. Baracka C. A. (1995). Lysophosphatidic acid induction of transforming growth factors α and β: modulation of proliferation and differentiation in cultured human keratinocytes and mouse skin. *Exp. Cell Res.* 216 51–64. 10.1006/excr.1995.1007 7813633

[B187] Piotrowska A. Wierzbicka J. Żmijewski M. A. (2016). Vitamin D in the skin physiology and pathology. *Acta Biochim. Pol.* 63 1104–1129. 10.18388/abp.2015_1104 26824295

[B188] Proksch E. Brandner J. M. Jensen J. (2008). The skin: an indispensable barrier. *Exp. Dermatol.* 17 1063–1072. 10.1111/j.1600-0625.2008.00786.x 19043850

[B189] Pucci M. Pasquariello N. Battista N. Tommaso M. D. Rapino C. Fezza F. (2012). Endocannabinoids stimulate human melanogenesis *via* Type-1 cannabinoid receptor*. *J. Biol. Chem.* 287 15466–15478. 10.1074/jbc.m111.314880 22431736 PMC3346111

[B190] Puhvel S. M. Reisner R. M. Sakamoto M. (1975). Analysis of lipid composition of isolated human sebaceous gland homogenates after incubation with cutaneous bacteria. thin-layer chromatography. *J. Invest Dermatol.* 64 406–411. 10.1111/1523-1747.ep12512337 237966

[B191] Reines I. Kietzmann M. Mischke R. Tschernig T. Lüth A. Kleuser B. (2009). Topical application of Sphingosine-1-Phosphate and FTY720 attenuate allergic contact dermatitis reaction through inhibition of dendritic cell migration. *J. Invest. Dermatol.* 129 1954–1962. 10.1038/jid.2008.454 19194476

[B192] Rhodes L. E. Belgi G. Parslew R. McLoughlin L. Clough G. F. Friedmann P. S. (2001). Ultraviolet-B-Induced erythema is mediated by nitric oxide and prostaglandin E2 in combination. *J. Invest. Dermatol.* 117 880–885. 10.1046/j.0022-202x.2001.01514.x 11676827

[B193] Rhodes L. E. Gledhill K. Masoodi M. Haylett A. K. Brownrigg M. Thody A. J. (2009). The sunburn response in human skin is characterized by sequential eicosanoid profiles that may mediate its early and late phases. *FASEB J.* 23 3947–3956. 10.1096/fj.09-136077 19584301 PMC2791058

[B194] Rivier M. Safonova I. Lebrun P. Michel S. Griffiths C. E. M. Ailhaud G. (1998). Differential expression of peroxisome proliferator-activated receptor subtypes during the differentiation of human keratinocytes. *J. Invest. Dermatol.* 111 1116–1121. 10.1046/j.1523-1747.1998.00439.x 9856826

[B195] Rivier M. Safonova I. Michel S. Castiel I. Ailhaud G. (2000). Peroxisome proliferator-activated receptor-α enhances lipid metabolism in a skin equivalent model. *J. Invest. Dermatol.* 114 681–687. 10.1046/j.1523-1747.2000.00939.x 10733673

[B196] Robbiani D. F. Finch R. A. Jäger D. Muller W. A. Sartorelli A. C. Randolph G. J. (2000). The leukotriene C4 transporter MRP1 regulates CCL19 (MIP-3β, ELC)-dependent mobilization of dendritic cells to lymph nodes. *Cell* 103 757–768. 11114332 10.1016/s0092-8674(00)00179-3

[B197] Rognoni E. Watt F. M. (2018). Skin cell heterogeneity in development, wound healing, and Cancer. *Trends Cell Biol.* 28 709–722. 10.1016/j.tcb.2018.05.002 29807713 PMC6098245

[B198] Rosse C. Linch M. Kermorgant S. Cameron A. J. M. Boeckeler K. Parker P. J. (2010). PKC and the control of localized signal dynamics. *Nat. Rev. Mol. Cell Biol.* 11 103–112. 10.1038/nrm2847 20094051

[B199] Rothman S. Smijanic A. M. Weitkamp A. W. (1946). Mechanism of spontaneous cure in puberty of ringworm of the scalp. *Science* 104 201–203. 10.1126/science.104.2696.201 17743940

[B200] Russell L. E. Harrison W. J. Bahta A. W. Zouboulis C. C. Burrin J. M. Philpott M. P. (2007). Characterization of liver X receptor expression and function in human skin and the pilosebaceous unit. *Exp. Dermatol.* 16 844–852. 10.1111/j.1600-0625.2007.00612.x 17845217

[B201] Sado-Kamdem S. L. Vannini L. Guerzoni M. E. (2009). Effect of α-linolenic, capric and lauric acid on the fatty acid biosynthesis in *Staphylococcus aureus*. *Int. J. Food Microbiol.* 129 288–294. 10.1016/j.ijfoodmicro.2008.12.010 19168249

[B202] Saito H. Tomioka H. Yoneyama T. (1984). Growth of group IV mycobacteria on medium containing various saturated and unsaturated fatty acids. *Antimicrob Agents Chemother* 26 164–169. 10.1128/aac.26.2.164 6486760 PMC284112

[B203] Samsonov A. V. Mihalyov I. Cohen F. S. (2001). Characterization of cholesterol-sphingomyelin domains and their dynamics in bilayer membranes. *Biophys. J.* 81 1486–1500. 11509362 10.1016/S0006-3495(01)75803-1 PMC1301627

[B204] Sato J. Denda M. Nakanishi J. Nomura J. Koyama J. (1998). Cholesterol sulfate inhibits proteases that are involved in desquamation of stratum corneum. *J. Invest. Dermatol.* 111 189–193. 10.1046/j.1523-1747.1998.00244.x 9699715

[B205] Sauer B. Vogler R. Wenckstern H. von, Fujii M. Anzano M. B. (2004). Involvement of smad signaling in sphingosine 1-Phosphate-mediated biological responses of keratinocytes*. *J. Biol. Chem.* 279 38471–38479. 10.1074/jbc.m313557200 15247277

[B206] Schade H. Marchionini A. (1928). Der Säuremantel der Haut (Nach Gaskettenmessungen). *Klin Wochenschr* 7 12–14. 10.1007/bf01711684

[B207] Scheimann L. G. Knox G. Sher D. Rothman S. (1960). The role of bacteria in the formation of free fatty acids on the human skin surface. *J. Invest. Dermatol.* 34 171–174. 10.1038/jid.1960.23 14442581

[B208] Schmitt T. Lange S. Sonnenberger S. Dobner B. Demé B. Langner A. (2019). The long periodicity phase (LPP) controversy part I: the influence of a natural-like ratio of the CER[EOS] analogue [EOS]-br in a CER[NP]/[AP] based stratum corneum modelling system: a neutron diffraction study. *Biochim. Biophys. Acta* 1861 306–315. 10.1016/j.bbamem.2018.06.008 29924985

[B209] Schmuth M. Haqq C. M. Cairns W. J. Holder J. C. Dorsam S. Chang S. (2004). Peroxisome proliferator-activated receptor (PPAR)-β/δ stimulates differentiation and lipid accumulation in keratinocytes. *J. Invest. Dermatol.* 122 971–983. 10.1111/j.0022-202x.2004.22412.x 15102088

[B210] Schüppel M. Kürschner U. Kleuser U. Schäfer-Korting M. Kleuser B. (2008). Sphingosine 1-Phosphate restrains insulin-mediated keratinocyte proliferation *via* inhibition of Akt through the S1P2 receptor subtype. *J. Invest. Dermatol.* 128 1747–1756. 10.1038/sj.jid.5701259 18219276

[B211] Seitz A. P. Schumacher F. Baker J. Soddemann M. Wilker B. Caldwell C. C. (2019). Sphingosine-coating of plastic surfaces prevents ventilator-associated pneumonia. *J. Mol. Med.* 97 1195–1211. 10.1007/s00109-019-01800-1801 31222488 PMC6647234

[B212] Seitz C. S. Freiberg R. A. Hinata K. Khavari P. A. (2000). NF-κB determines localization and features of cell death in epidermis. *J. Clin. Invest.* 105 253–260. 10.1172/jci7630 10675350 PMC377441

[B213] Selby C. C. (1957). An electron microscope study of thin sections of human skin II. superficial cell layers of footpad epidermis 1. *J. Invest. Dermatol.* 29 131–149. 10.1038/jid.1957.80 13475953

[B214] Sheu C. W. Freese E. (1972). Effects of fatty acids on growth and envelope proteins of *Bacillus subtilis*. *J. Bacteriol.* 111 516–524. 10.1128/jb.111.2.516-524.1972 4626502 PMC251313

[B215] Shinomura T. Asaoka Y. Oka M. Yoshida K. Nishizuka Y. (1991). Synergistic action of diacylglycerol and unsaturated fatty acid for protein kinase C activation: its possible implications. *Proc. Natl. Acad. Sci. U S A.* 88 5149–5153. 10.1073/pnas.88.12.5149 1905018 PMC51829

[B216] Simons K. Sampaio J. L. (2011). Membrane organization and lipid rafts. *Cold Spring Harb. Perspect. Biol.* 3:a004697. 10.1101/cshperspect.a004697 21628426 PMC3179338

[B217] Školová B. Kováčik A. Tesař O. Opálka L. Vávrová K. (2017). Phytosphingosine, sphingosine and dihydrosphingosine ceramides in model skin lipid membranes: permeability and biophysics. *BBA - Biomembranes* 1859 824–834. 10.1016/j.bbamem.2017.01.019 28109750

[B218] Smita K. Kumar V. S. Premendran J. S. (2007). Anandamide: an update. *Fundam. Clin. Pharm.* 21 1–8. 10.1111/j.1472-8206.2006.00454.x 17227440

[B219] Spik I. Brénuchon C. Angéli V. Staumont D. Fleury S. Capron M. (2005). Activation of the Prostaglandin D2 receptor DP2/CRTH2 increases allergic inflammation in mouse. *J. Immunol.* 174 3703–3708. 10.4049/jimmunol.174.6.3703 15749909

[B220] Stahley S. N. Saito M. Faundez V. Koval M. Mattheyses A. L. Kowalczyk A. P. (2014). Desmosome assembly and disassembly are membrane raft-dependent. *PLoS One* 9:e87809. 10.1371/journal.pone.0087809 24498201 PMC3907498

[B221] Stewart M. E. Downing D. T. (1991). Chemistry and function of mammalian sebaceous lipids. *Adv. Lipid Res.* 24 263–301. 10.1016/b978-0-12-024924-4.50013-50014 1763714

[B222] Sugiura T. Kishimoto S. Oka S. Gokoh M. (2006). Biochemistry, pharmacology and physiology of 2-arachidonoylglycerol, an endogenous cannabinoid receptor ligand. *Prog. Lipid Res.* 45 405–446. 10.1016/j.plipres.2006.03.003 16678907

[B223] Sumitomo A. Siriwach R. Thumkeo D. Ito K. Nakagawa R. Tanaka N. (2019). LPA induces keratinocyte differentiation and promotes skin barrier function through the LPAR1/LPAR5-RHO-ROCK-SRF Axis. *J. Invest. Dermatol.* 139 1010–1022. 10.1016/j.jid.2018.10.034 30447238

[B224] Suzuki Y. Nomura J. Hori J. Koyama J. Takahashi M. Horii I. (1993). Detection and characterization of endogenous protease associated with desquamation of stratum corneum. *Arch. Dermatol. Res.* 285 372–377. 10.1007/bf00371839 8215586

[B225] Swartzendruber D. C. Wertz P. W. Kitko D. J. Madison K. C. Downing D. T. (1989). Molecular models of the intercellular lipid lamellae in mammalian stratum corneum. *J. Invest. Dermatol.* 92 251–257. 10.1111/1523-1747.ep12276794 2918233

[B226] Swartzendruber D. C. Wertz P. W. Madison K. C. Downing D. T. (1987). Evidence that the corneocyte has a chemically bound lipid envelope. *J. Invest. Dermatol.* 88 709–713. 10.1111/1523-1747.ep12470383 3585054

[B227] Szymański Ł Skopek R. Palusińska M. Schenk T. Stengel S. Lewicki S. (2020). Retinoic acid and its derivatives in skin. *Cells* 9:2660. 10.3390/cells9122660 33322246 PMC7764495

[B228] Takigawa H. Nakagawa H. Kuzukawa M. Mori H. Imokawa G. (2005). Deficient production of hexadecenoic acid in the skin is associated in part with the vulnerability of atopic dermatitis patients to colonization by *Staphylococcus aureus*. *Dermatology* 211 240–248. 10.1159/000087018 16205069

[B229] Theiner G. Gessner A. Lutz M. B. (2006). The mast cell mediator PGD2 suppresses IL-12 release by dendritic cells leading to Th2 polarized immune responses in vivo. *Immunobiology* 211 463–472. 10.1016/j.imbio.2006.05.020 16920486

[B230] Thormar H. Hilmarsson H. (2007). The role of microbicidal lipids in host defense against pathogens and their potential as therapeutic agents. *Chem. Phys. Lipids* 150 1–11. 10.1016/j.chemphyslip.2007.06.220 17686469

[B231] Tober K. L. Thomas-Ahner J. M. Maruyama T. Oberyszyn T. M. (2007). Possible cross-regulation of the E prostanoid receptors. *Mol. Carcinogen.* 46 711–715. 10.1002/mc.20347 17538953

[B232] Tóth B. I. Dobrosi N. Dajnoki A. Czifra G. Oláh A. Szöllősi A. G. (2011). Endocannabinoids modulate human epidermal keratinocyte proliferation and survival *via* the sequential engagement of cannabinoid Receptor-1 and transient receptor potential Vanilloid-1. *J. Invest. Dermatol.* 131 1095–1104. 10.1038/jid.2010.421 21248768

[B233] Tsuji K. Mitsutake S. Yokose U. Sugiura M. Kohama T. Igarashi Y. (2008). Role of ceramide kinase in peroxisome proliferator-activated receptor beta-induced cell survival of mouse keratinocytes. *FEBS J.* 275 3815–3826. 10.1111/j.1742-4658.2008.06527.x 18565104

[B234] Uchida Y. (2014). Ceramide signaling in mammalian epidermis. *Biochim. Biophys. Acta* 1841 453–462. 10.1016/j.bbalip.2013.09.003 24055887 PMC3943494

[B235] Uchida Y. Park K. (2021). Ceramides in skin health and disease: an update. *Am. J. Clin. Dermatol.* 22 853–866. 10.1007/s40257-021-00619-612 34283373

[B236] Uchida Y. Nardo A. D. Collins V. Elias P. M. Holleran W. M. (2003). De novo ceramide synthesis participates in the ultraviolet B irradiation-induced apoptosis in undifferentiated cultured human keratinocytes. *J. Invest. Dermatol.* 120 662–669. 10.1046/j.1523-1747.2003.12098.x 12648232

[B237] Vahlquist A. Törmä H. (2020). Ichthyosis: a road model for skin research. *Acta Dermato Venereol.* 100:adv00097. 10.2340/00015555-13433 PMC9128938 32147743

[B238] van Corven E. J. van Rijswijk A. Jalink K. van der Bend R. L. van Blitterswijk W. J. Moolenaar W. H. (1992). Mitogenic action of lysophosphatidic acid and phosphatidic acid on fibroblasts. dependence on acyl-chain length and inhibition by suramin. *Biochem. J.* 281 163–169. 10.1042/bj2810163 1731751 PMC1130655

[B239] van Meer G. Voelker D. R. Feigenson G. W. (2008). Membrane lipids: where they are and how they behave. *Nat. Rev. Mol. Cell Biol.* 9 112–124. 10.1038/nrm2330 18216768 PMC2642958

[B240] van Smeden J. Hoppel L. Heijden R. van der, Hankemeier T. Vreeken R. J. (2011). LC/MS analysis of stratum corneum lipids: ceramide profiling and discovery. *J. Lipid Res.* 52 1211–1221. 10.1194/jlr.m014456 21444759 PMC3090242

[B241] Vietri Rudan M. Mishra A. Klose C. Eggert U. S. Watt F. M. (2020). Human epidermal stem cell differentiation is modulated by specific lipid subspecies. *Proc. Natl. Acad. Sci. U S A.* 117:202011310. 10.1073/pnas.2011310117 32843345 PMC7486749

[B242] Vogler R. Sauer B. Kim D.-S. Schäfer-Korting M. Kleuser B. (2003). Sphingosine-1-Phosphate and its potentially paradoxical effects on critical parameters of cutaneous wound healing. *J. Invest. Dermatol.* 120 693–700. 10.1046/j.1523-1747.2003.12096.x 12648236

[B243] Wacnik P. W. Luhr K. M. Hill R. H. Ljunggren H.-G. Kristensson K. Svensson M. (2008). Cannabinoids affect dendritic cell (DC) potassium channel function and modulate DC T Cell stimulatory capacity. *J. Immunol.* 181 3057–3066. 10.4049/jimmunol.181.5.3057 18713976

[B244] Wakita H. Matsushita K. Nishimura K. Tokura Y. Furukawa F. Takigawa M. (1998). Sphingosylphosphorylcholine stimulates proliferation and upregulates cell surface-associated plasminogen activator activity in cultured human keratinocytes. *J. Invest. Dermatol.* 110 253–258. 10.1046/j.1523-1747.1998.00120.x 9506444

[B245] Wakita H. Tokura Y. Yagi H. Nishimura K. Furukawa F. Takigawa M. (1994). Keratinocyte differentiation is induced by cell-permeant ceramides and its proliferation is promoted by sphingosine. *Arch. Dermatol. Res.* 286 350–354. 10.1007/bf00402228 7979551

[B246] Wang J. Ueda N. (2009). Biology of endocannabinoid synthesis system. *Prostaglandins Other Lipid Mediat.* 89 112–119. 10.1016/j.prostaglandins.2008.12.002 19126434

[B247] Wanner R. Peiser M. Wittig B. (2004). Keratinocytes rapidly readjust ceramide-sphingomyelin homeostasis and contain a phosphatidylcholine-sphingomyelin transacylase. *J. Invest. Dermatol.* 122 773–782. 10.1111/j.0022-202x.2004.22340.x 15086565

[B248] Watt F. M. (2014). Mammalian skin cell biology: at the interface between laboratory and clinic. *Science* 346 937–940. 10.1126/science.1253734 25414300

[B249] Watt F. M. Collins C. A. (2008). Role of β-catenin in epidermal stem cell expansion, lineage selection, and Cancer. *Cold Spring Harb. Sym.* 73 503–512. 10.1101/sqb.2008.73.011 19022747

[B250] Watt F. M. Mattey D. L. Garrod D. R. (1984). Calcium-induced reorganization of desmosomal components in cultured human keratinocytes. *J. Cell Biol.* 99 2211–2215. 10.1083/jcb.99.6.2211 6209289 PMC2113584

[B251] Weitkamp A. W. Smiljanic A. M. Rothman S. (1947). The free fatty acids of human hair fat. *J. Am. Chem. Soc.* 69 1936–1939. 10.1021/ja01200a027 20255282

[B252] Wertz P. (2018). Epidermal lamellar granules. *Skin Pharmacol. Phys.* 31 262–268. 10.1159/000491757 30110701

[B253] Wertz P. W. (1996). The nature of the epidermal barrier: biochemical aspects. *Adv. Drug Deliver Rev.* 18 283–294. 10.1016/0169-409x(95)00077-k

[B254] Wertz P. W. (2000). Lipids and barrier function of the skin. *Acta Derm-venereol.* 80 7–11. 10.1080/000155500750042790 10884933

[B255] Wertz P. W. Downing D. T. (1987). Covalently bound ω-hydroxyacylsphingosine in the stratum corneum. *Biochim. Biophys. Acta* 917 108–111. 3790600 10.1016/0005-2760(87)90290-6

[B256] Wertz P. W. Downing D. T. (1990). Free sphingosine in human epidermis. *J. Invest. Dermatol.* 94 159–161. 10.1111/1523-1747.ep12874122 2299191

[B257] Wertz P. W. van den Bergh B. (1998). The physical, chemical and functional properties of lipids in the skin and other biological barriers. *Chem. Phys. Lipids* 91 85–96. 10.1016/s0009-3084(97)00108-104 9569614

[B258] Wertz P. W. Downing D. T. Freinkel R. K. Traczyk T. N. (1984). Sphingolipids of the stratum corneum and lamellar granules of fetal rat epidemis. *J. Invest. Dermatol.* 83 193–195. 10.1111/1523-1747.ep12263553 6470524

[B259] Wertz P. W. Schwartzendruber D. C. Madison K. C. Downing D. T. (1987). Composition and morphology of epidermal cyst lipids. *J. Invest. Dermatol.* 89 419–425. 10.1111/1523-1747.ep12471781 3668284

[B260] Wertz P. Downing D. (1982). Glycolipids in mammalian epidermis: structure and function in the water barrier. *Science* 217 1261–1262. 10.1126/science.7112128 7112128

[B261] Wilkinson D. I. Karasek M. A. (1966). Skin lipids of a normal and a mutant (Asebic) mouse strain. *J. Invest. Dermatol.* 47 449–455. 10.1038/jid.1966.168 5924301

[B262] Wille J. J. Kydonieus A. (2003). Palmitoleic acid isomer (C16:1Δ6) in human skin sebum is effective against gram-positive bacteria. *Skin Pharmacol. Phys.* 16 176–187. 10.1159/000069757 12677098

[B263] Wojtczak L. Załuska H. (1967). The inhibition of translocation of adenine nucleotides through mitochondrial membranes by oleate. *Biochem. Biophy. Res. Commun.* 28 76–81. 10.1016/0006-291x(67)90409-3 4227821

[B264] Yahagi S. Koike M. Okano Y. Masaki H. (2011). Lysophospholipids improve skin moisturization by modulating of calcium-dependent cell differentiation pathway. *Int. J. Cosmetic Sci.* 33 251–256. 10.1111/j.1468-2494.2010.00625.x 21272041

[B265] Zheng C. J. Yoo J.-S. Lee T.-G. Cho H.-Y. Kim Y.-H. Kim W.-G. (2005). Fatty acid synthesis is a target for antibacterial activity of unsaturated fatty acids. *FEBS Lett.* 579 5157–5162. 10.1016/j.febslet.2005.08.028 16146629

[B266] Zhu A. J. Watt F. M. (1999). beta-catenin signalling modulates proliferative potential of human epidermal keratinocytes independently of intercellular adhesion. *Development* 126 2285–2298. 10.1242/dev.126.10.2285 10207152

[B267] Ziboh V. A. Miller C. C. Cho Y. (2000). Metabolism of polyunsaturated fatty acids by skin epidermal enzymes: generation of antiinflammatory and antiproliferative metabolites. *Am. J. Clin. Nutrition* 71 361s–366s. 10.1093/ajcn/71.1.361s 10617998

[B268] Zouboulis C. C. (2004). Acne and sebaceous gland function. *Clin. Dermatol.* 22 360–366. 10.1016/j.clindermatol.2004.03.004 15556719

[B269] Zouboulis C. C. Seltmann H. Orfanos C. E. Neitzel H. (1999). Establishment and characterization of an immortalized human sebaceous gland cell line (SZ95)1. *J. Invest. Dermatol.* 113 1011–1020. 10.1046/j.1523-1747.1999.00771.x 10594745

[B270] Züllig T. Trötzmüller M. Köfeler H. C. (2020). Lipidomics from sample preparation to data analysis: a primer. *Anal. Bioanal. Chem.* 412 2191–2209. 10.1007/s00216-019-02241-y 31820027 PMC7118050

